# Moderating effect of cognitive reserve on brain integrity and cognitive performance

**DOI:** 10.3389/fnagi.2022.1018071

**Published:** 2022-11-03

**Authors:** Monica E. Nelson, Britney M. Veal, Ross Andel, Julie Martinkova, Katerina Veverova, Hana Horakova, Zuzana Nedelska, Jan Laczó, Martin Vyhnalek, Jakub Hort

**Affiliations:** ^1^School of Aging Studies, University of South Florida, Tampa, FL, United States; ^2^Edson College of Nursing and Health Innovation, Arizona State University, Phoenix, AZ, United States; ^3^Department of Neurology, Second Faculty of Medicine, Charles University and Motol University Hospital, Prague, Czechia

**Keywords:** executive control, attention/working memory, language, MRI, visuospatial skills

## Abstract

**Background:**

Dementia syndrome is one of the most devastating conditions in older adults. As treatments to stop neurodegeneration become available, accurate and timely diagnosis will increase in importance. One issue is that cognitive performance sometimes does not match the corresponding level of neuropathology, affecting diagnostic accuracy. Cognitive reserve (CR), which can preserve cognitive function despite underlying neuropathology, explains at least some variability in cognitive performance. We examined the influence of CR proxies (education and occupational position) on the relationship between hippocampal or total gray matter volume and cognition.

**Methods:**

We used data from the Czech Brain Aging Study. Participants were clinically confirmed to be without dementia (*n* = 457, including subjective cognitive decline and amnestic mild cognitive impairment) or with dementia syndrome (*n* = 113).

**Results:**

For participants without dementia, higher education magnified the associations between (a) hippocampal volume and executive control (*b* = 0.09, *p* = 0.033), (b) total gray matter volume and language (*b* = 0.12, *p* < 0.001), and (c) total gray matter volume and memory (*b* = 0.08, *p* = 0.018). Similarly, higher occupational position magnified the association between total gray matter volume and (a) attention/working memory (*b* = 0.09, *p* = 0.009), (b) language (*b* = 0.13, *p* = 0.002), and (c) memory (*b* = 0.10, *p* = 0.013). For participants with dementia, the associations between hippocampal (*b* = –0.26, *p* = 0.024) and total gray matter (*b* = –0.28, *p* = 0.024) volume and visuospatial skills decreased in magnitude with higher education.

**Conclusion:**

We found that the association between brain volume and cognitive performance varies based on CR, with greater CR related to a stronger link between brain volume and cognition before, and a weaker link after, dementia diagnosis.

## Introduction

Alzheimer’s disease (AD) is the most prevalent cause of dementia syndrome in older adults (Alzheimer’s Association, 2022). The pathophysiological mechanisms thought to cause AD include impaired amyloid-beta and tau protein metabolism and neurodegeneration leading to regional and whole brain atrophy (according to the amyloid cascade hypothesis; Hardy and Higgins, 1992; Karran et al., 2011; Jack and Holtzman, 2013; Jack et al., 2013); however, it is known that clinical progression of the disease does not always align with levels of neuropathology load. Therefore, there can be a dichotomy between expected and observed cognitive performance based on level of neuropathology which raises the question of what additional factors may be contributing to the preservation of cognitive functions in older adults. Stern et al. (2020) discuss the concept of resilience, which is related to brain reserve, brain maintenance, and cognitive reserve, as an explanation for this dichotomy.

Cognitive reserve is an active mechanism thought to be influenced by lifetime experiences and achievements and explains better cognitive performance in the presence of neuropathology (Stern et al., 2020). Cognitive reserve is typically measured through sociobehavioral proxies (e.g., education or occupation) which are thought to contribute to reserve (Stern, 2002, 2009). Stern et al. (2020) suggest that an effective way to test the cognitive reserve hypothesis is through examining whether measures of reserve moderate the association between brain health and cognition.

Some studies have assessed how markers of cognitive reserve, such as education or occupation, moderate the relationship between AD-related neuropathology and cognitive outcomes but with discrepant findings. For example, in healthy older adults, higher educational attainment was associated with a *weaker* negative relationship between positron emission tomography (PET)-assessed amyloid-beta load and episodic memory performance (Joannette et al., 2020). However, higher education was also associated with a *stronger* positive relationship between hippocampal volume and delayed recall in healthy older adults (O’Shea et al., 2018). Similar divergent results have emerged in samples that included participants with cognitive impairment. For example, in a Dutch sample of older adults from a memory clinic that included participants with subjective cognitive impairment (SCI), mild cognitive impairment (MCI), and AD dementia, there was a *weaker* positive relationship between medial temporal lobe volume and global cognitive abilities (i.e., greater volume/less atrophy related to better cognition) in more educated participants (Staekenborg et al., 2020). However, a study using data from a memory clinic in South Korea, which included participants with normal cognitive function, MCI, and dementia, reported that less right temporoparietal tau pathology and greater cortical thickness of the left inferior temporal gyrus were related to better memory performance—these relationships were *stronger* for individuals with higher cognitive reserve (a combination of education and work complexity) (Lee et al., 2019). Clearly, more research is needed to disentangle discrepancies between studies investigating cognitive reserve, brain health, and cognitive outcomes, especially within (rather than across) subgroups with the same cognitive status.

It is possible that these discrepant findings may result from the presence of a non-linear relationship between cognitive reserve, brain health markers, and cognitive outcomes. Evidence suggests that cognitive reserve interacts with cognitive performance and brain health differentially depending on the cognitive status of the sample being studied (e.g., healthy versus pathological aging; Menardi et al., 2018). Specifically, the interaction between brain health and cognition transitions from protective in healthy aging (i.e., higher cognitive reserve associated with better brain health) to compensatory in pathological aging (i.e., higher cognitive reserve associated with greater pathology; Menardi et al., 2018), but in both situations, higher cognitive reserve is associated with better cognitive performance. However, this explanation may be too simple since divergent findings regarding the moderating effect of cognitive reserve on brain health-cognition relationships have been found in both healthy and pathological aging.

Building on past research (O’Shea et al., 2018; Lee et al., 2019; Joannette et al., 2020; Staekenborg et al., 2020), we investigated how two common proxies of cognitive reserve (education and occupational position) moderated the relationship between brain health, represented by hippocampal and total gray matter volume, and cognition in participants without dementia (i.e., subjective cognitive decline [SCD] and amnestic MCI [aMCI]) and with dementia syndrome. We modeled our approach after Joannette et al. (2020) who identified regions of significant moderations utilizing the Johnson–Neyman technique (Johnson and Fay, 1950), but with significant moderations probed via pre-defined groupings of cognitive reserve (Joannette et al., 2020). Since we used a sample from a memory clinic with variation in levels of clinical impairment, we may be able to detect at what point the relationship between brain volume and cognition becomes affected by markers of cognitive reserve. To extend past research that used only memory (O’Shea et al., 2018; Joannette et al., 2020) or general cognitive abilities (Staekenborg et al., 2020) as the cognitive outcome, we assessed a full range of cognitive domains in analyses.

## Materials and methods

### Participants

The Czech Brain Aging Study (CBAS) is a longitudinal cohort study of patients in the Memory Clinic in Motol University Hospital and Charles University, Prague, Czech Republic (Sheardova et al., 2019). The participants were referred to the Memory Clinic by general practitioners, neurologists, or psychiatrists for self- and/or informant-reported cognitive complaints. The presented cross-sectional study utilized the cognitive testing and neuroimaging data from these participants, though other studies on laboratory and genetic results and spatial navigation exist. All participants provided informed consent. The Institutional Review Board of Motol University Hospital continually approves this research project.

A total of 873 individuals had at least one magnetic resonance imaging (MRI) scan, forming the initial sample. Participants with greater than 6 months between their MRI scan and neuropsychological tests (*n* = 33), missing information on education (*n* = 9), occupational position (*n* = 77), or diagnosis (*n* = 22), who had a main lifetime occupation in the armed forces (*n* = 5), who had non-amnestic MCI (*n* = 84), or completed less than 50% of neuropsychological tests used to create each of the cognitive domains (*n* = 19) were dropped. To limit the sample to participants with mild dementia, participants diagnosed with dementia who did not have information about their Mini Mental State Exam (MMSE) or who had a score less than 18 were dropped (*n* = 54). Therefore, the final analytic sample included 570 participants who were diagnosed without dementia (*n* = 457; including SCD [*n* = 149] and aMCI [*n* = 308]) or with dementia syndrome (*n* = 113). We included aMCI specifically since (a) it has been shown to relate more strongly to dementia progression than non-amnestic MCI (Peltz et al., 2011; Michaud et al., 2017), (b) it is the most common type of MCI, and (c) it is more likely associated with a neurodegenerative cause compared to non-amnestic MCI (Petersen et al., 2009).

Participants without dementia included individuals with clinical diagnoses of SCD or aMCI. SCD was defined as having normal objective cognitive performance, self-reported persistent cognitive complaints within the last 5 years in comparison with a previously normal status and being unrelated to an acute event, and having a Clinical Dementia Rating (CDR) global score of ≤ 0.5 (Jessen et al., 2014). aMCI was defined as scoring ≥ 1.5 standard deviations below age- and education-adjusted neuropsychological test means in at least one of the memory tests, a CDR score ≤ 0.5, and cognitive complaints reported by the patient or a reliable informant (Petersen, 2004). Although aMCI was based mainly on neuropsychological tests, agreement within a multidisciplinary clinical team was required for the formal diagnosis, including a cognitive neurologist, clinical neuropsychologist, and psychometricians, taking into account the nature of the cognitive complaints and the patient’s medical history. To maintain parsimonious presentation of the results, we combined the two groups indicative of cognitive impairment (but without dementia)—SCD and aMCI. The decision to group SCD and aMCI is supported by the fact that individuals with SCD and aMCI are presumed to be on a cognitive continuum with at least some overlap whereby the two diagnoses have a tendency to fluctuate over time.

Dementia syndrome was diagnosed based on a consensus panel (Sheardova et al., 2019) using the Diagnostic and Statistical Manual of Mental Disorders: Text Revision (DSM-IV-TR) guidelines (American Psychiatric Association, 2000). The progression to dementia and the etiology of dementia syndrome was established during the regular consensus meetings of neurologists and neuropsychologists. The diagnosis was mainly based on clinical history reported by the patient and the caregiver, neurological examination, and neuropsychological assessment. Brain MRI and PET scans were also considered when available. Most participants diagnosed with dementia syndrome had either AD (42%) or mixed dementia (40%) as their etiology, with other etiologies (e.g., vascular dementia, frontotemporal dementia, dementia with Lewy bodies; 15%) or unavailable etiologies (4%) present in the remainder of the sample. The etiology of dementia syndrome was based on the current clinical criteria for probable AD (McKhann et al., 2011), probable vascular dementia (Román et al., 1993), probable dementia with Lewy bodies (McKeith et al., 2017), or probable behavioral variant of frontotemporal dementia (Rascovsky et al., 2011).

### Measures

#### Cognitive domains

Neuropsychological tests were used to create cognitive domains by standardizing scores on each test then averaging the individual tests into a composite variable. Standardization was completed separately for participants with and without dementia; thus, participants’ performance on these tests would be compared against other participants in the same diagnostic group.

##### Attention/working memory

This domain (α = 0.78) was assessed with four tests. We used Trail Making Test – Part A (TMT-A) with a maximum time of 180 s (Bezdicek et al., 2012). Participants who were unable to complete it within the given time frame were scored as 181 s. For analyses the score was reversed. The other tests included: Digit Symbol Coding Test, Digit Span – Forward (Digits-F), and Digit Span – Backward (Digits-B) from the Wechsler Adult Intelligence Scale – Revised (Wechsler, 1997), assessed with sub-scores (i.e., number of correct items).

##### Executive control

This domain (α = 0.62) was assessed with two tests: Controlled Oral Word Association Test (COWAT) with letters N, K, and P (Czech Republic Version) (Nikolai et al., 2015), and Trail Making Test – Part B (TMT-B) (Bezdicek et al., 2012). The maximum time for completion of the TMT-B was 300 s. However, those who were unable to complete it within the given time frame were scored as 301 s. For analyses the score was reversed.

##### Language

This domain (α = 0.84) was assessed with four tests: Boston Naming Test (BNT) (Goodglass et al., 1983), Verbal Fluency Test – Vegetables (VFT-V), Verbal Fluency Test – Animals (VFT-A) (Nikolai et al., 2015), and the Similarities subtest from the Wechsler Adult Intelligence Scale – III (Wechsler, 1997).

##### Memory

This domain (α = 0.93) was assessed with four tests. Specifically, the Rey Auditory Verbal Learning Test – Immediate Recall (AVLT-I; Sum of Trials 1-5) and Delayed Recall (AVLT-D) (Bezdicek et al., 2014), Brief Visuospatial Memory Test – Revised Immediate Recall (BVMTR-I; Sum of Trials 1–3) and Delayed Recall (BVMTR-D) (Benedict et al., 1996), and the Uniform Data Set Logical Memory Immediate Recall Test (Log-I) and Delayed Recall Test (Log-D) (Nikolai et al., 2018). The Rey–Osterrieth Complex Figure Test Recall was a test of non-verbal memory (Osterrieth, 1944). The AVLT, BVMTR, and Log included both immediate and delayed components in one exam.

##### Visuospatial skills

This domain (α = 0.77) was assessed with two tests: Clock Drawing Test (Mazancova et al., 2017) (Cohen scoring system) and the Rey-Osterrieth Complex Figure Test Copy (Meyers and Meyers, 1995).

#### Magnetic resonance imaging

MRI scans were acquired on a 1.5T scanner (Avanto; Siemens, Erlangen, Germany) using T1-weighted three-dimensional high-resolution magnetization-prepared rapid acquisition with gradient echo (MPRAGE) sequence with the following parameters: TR/TE/TI = 2000/3.08/1100 ms, flip angle 15°, 192 continuous partitions, slice thickness 1.0 mm, and in-plane resolution 1 mm. Scans were visually examined by a neuroradiologist, blinded to the diagnosis, for sufficient technical quality and the absence of structural findings contradicting eligibility (Nedelska et al., 2012; Kerbler et al., 2015). MRI volumetry was processed using Freesurfer automated package (v5.3.0^1^; Fischl et al., 2002). The current analyses focused on total hippocampal volume, which was calculated as the sum of the right and left hippocampal volumes, and total gray matter volume, both measured in cubic millimeters. Hippocampal volume and total gray matter volume were adjusted for estimated total intracranial volume (eTIV) to adjust for differences in head size (Voevodskaya et al., 2014). For analyses, eTIV-adjusted volumes were converted to *z*-scores based on diagnostic groups (i.e., participants without and with dementia).

#### Cognitive reserve proxies

Two cognitive reserve proxies were used in the current study. Education was measured as years of formal education (range: 8–27 years). Occupational position was coded by two independent raters according to the 2008 International Standard Classification of Occupations (ISCO-08; International Labour Office, 2012). The raters (JM, KV) were familiar with the data and native speakers of Czech language. The initial agreement was 87.5%. Coding for the remaining 12.5% of occupations was finalized during a consensus meeting between the two raters. Occupational position was coded based on main lifetime occupation; where unavailable (34.6%), last occupation was used. Participants were classified into one of the 10 categories represented by ISCO-08. Lower scores corresponded to higher occupational positions (1: managers, 2: professionals, 3: technicians and associate professionals, 4: clerical support workers, 5: services and sales workers, 6: skilled agricultural, forestry, and fishery workers, 7: craft and related trades workers, 8: plant and machine operators and assemblers, 9: elementary occupations, 0: armed forces workers). Due to the varied occupational positions that armed forces workers could align with, participants whose main lifetime occupation corresponded to armed forces workers were dropped from analyses. For analytic purposes, the ISCO-08 score was recoded such that higher scores were associated with higher occupational positions. We assessed the relative strength of education and occupation as cognitive reserve proxies by conducting analyses using standardized variables (*z*-scores). For interpretation of interactions, we graphed the associations between brain volume and cognition for individuals with high (+1 SD from mean) and low (–1 SD from mean) cognitive reserve.

#### Covariates

Age (years), sex (male or female), and depressive symptoms as measured by the Geriatric Depression Scale 15-item version (Yesavage et al., 1982) (GDS-15; cutoff ≥ 5) served as covariates.

### Statistical analyses

Descriptive statistics were used to describe the two groups. Independent sample *t*-tests or chi-square tests were used to assess differences between groups on the study variables. Separate regression analyses were conducted by diagnosis to examine the interrelation between brain volume (i.e., hippocampal or total gray matter volume), cognitive reserve proxies (education or occupational position), and cognition (five cognitive domains). Covariates included age, sex, and depressive symptoms. Since the relationship between depressive symptoms and cognitive performance is complex (Butters et al., 2008) (e.g., depression could be a risk factor for, consequence of, or reaction to cognitive impairment), we also conducted analyses not controlling for depressive symptoms to determine the stability of effects. Additionally, as sex has been shown to influence reserve or resilience effects (Sundermann et al., 2016; Ewers, 2020; Subramaniapillai et al., 2021; Pa et al., 2022), we also conducted analyses stratified by sex.

We examined whether the cognitive reserve proxy variables moderated the relationship between brain volume and cognition. Significant interactions were probed with the Johnson-Neyman technique (Johnson and Fay, 1950) to identify “regions of significance,” that is, points at which there were significant moderated relationships between brain volume and cognition (Joannette et al., 2020). Significant interactions between brain volume and cognitive reserve proxies were graphed with high and low reserve corresponding to ±1 SD from the mean, respectively. Since main lifetime occupation represents the longest exposure to work environment and is therefore more likely to contribute to cognitive reserve than last occupation, we conducted sensitivity analyses restricting our sample to only participants who had data available on main lifetime occupation. All analyses were conducted using SAS software, Version 9.4 of the SAS System for Windows (SAS Institute, Cary, NC, USA). Moderation analyses were conducted with the PROCESS Macro Version 3.4.1 in SAS (Hayes and Little, 2018). Significance was assessed with a two-tailed test at *p* < 0.05. In order to account for multiple comparisons, we used the Holm–Bonferroni Sequential Correction method (Holm, 1979) in a model-wise fashion such that the lowest *p*-value within a model was compared to the most stringent level of significance (*p* < 0.005) which was reduced iteratively for subsequent effects. Effects were considered significant if they fell below the level prespecified by the correction method.

## Results

Table 1 contains descriptive statistics for all study variables. Participants without dementia (i.e., SCD or aMCI) were significantly younger than participants with dementia. There was a greater frequency of women than men in the full sample, with no significant difference between groups. Participants without dementia had higher educational attainment and occupational position than participants with dementia. Depressive symptoms did not differ between groups. The diagnostic groups differed in terms of hippocampal volume, total gray matter volume, MMSE score, and each of the raw scores for the neuropsychological tests, as expected, such that brain volume was greater and cognition was better in participants without dementia compared to participants with dementia.

**TABLE 1:** Descriptive statistics of study variables for the full sample and by diagnosis.

	Full sample		No dementia		Dementia		
			
Variable	*M* or *N*	*SD* or %	*M* or *N*	*SD* or %	*M* or *N*	*SD* or %	*P-value* ^d^
Age	71.20	8.76	70.49	8.61	74.01	8.84	<0.001
Sex							0.182
Male	238	41.83%	197	43.20%	41	36.28%	
Female	331	58.17%	259	56.80%	72	63.72%	
Mini mental state exam	25.75	3.61	26.78	3.00	21.62	2.84	<0.001
GDS-15	3.98	3.23	3.88	3.14	4.35	3.55	0.172
Education, years	14.52	3.32	14.89	3.33	13.02	2.81	<0.001
Occupational position^a^	5.56	1.96	5.75	1.90	4.81	2.02	<0.001
Managers	46	8.07%	43	9.41%	3	2.65%	
Professionals	198	34.74%	172	37.64%	26	23.01%	
Technicians and associate professionals	114	20.00%	95	20.79%	19	16.81%	
Clerical support workers	75	13.16%	53	11.60%	22	19.47%	
Services and sales workers	59	10.35%	41	8.97%	18	15.93%	
Skilled agricultural, forestry, and fishery workers	5	0.88%	2	0.44%	3	2.65%	
Craft and related trades workers	43	7.54%	28	6.13%	15	13.27%	
Plant and machine operators and assemblers	20	3.51%	17	3.72%	3	2.65%	
Elementary occupations	10	1.75%	6	1.31%	4	3.54%	
Hippocampal volume, mm^3^	6771.92	1317.73	6960.89	1278.34	6007.65	1197.18	<0.001
Total gray matter volume, mm^3^	547462.41	64610.19	558187.74	61579.32	504086.52	58320.50	<0.001
**Attention/Working memory**							
Trail making test – Part A^b^	66.08	40.02	57.76	31.59	102.98	51.33	<0.001
Digit span – Forward^c^	8.38	2.15	8.60	2.18	7.46	1.75	<0.001
Digit span – Backward^c^	5.36	2.18	5.69	2.16	4.00	1.71	<0.001
Digit symbol coding test	30.54	12.74	33.14	11.88	19.11	9.77	<0.001
**Executive control**							
COWAT	34.46	14.15	36.91	13.71	24.14	10.98	<0.001
Trail making test – Part B^b^	148.42	76.31	140.95	71.34	225.33	83.08	<0.001
**Language**							
BNT – 60 item version	53.01	6.77	53.66	6.33	50.38	7.80	<0.001
VFT – Vegetables	9.62	3.87	10.27	3.87	6.80	2.32	<0.001
VFT – Animals	17.70	7.48	19.14	7.20	11.42	5.11	<0.001
Similarities subtest from WAIS-III	21.57	7.08	22.55	6.60	13.58	5.65	<0.001
**Memory**							
AVLT 1–5 (Immediate)	36.01	12.82	37.56	12.60	24.36	7.24	<0.001
BVMTR (Immediate)	10.10	10.08	12.03	10.13	2.28	4.56	<0.001
Logical memory (Immediate)	10.21	5.31	11.16	5.18	6.43	3.98	<0.001
AVLT 30 (Delayed)	5.39	4.45	5.91	4.38	1.47	2.71	<0.001
BVMTR (Delayed)	5.85	3.88	6.16	3.84	2.21	2.17	<0.001
Logical memory (Delayed)	7.25	6.07	8.33	6.05	2.86	3.77	<0.001
ROCFT recall	9.78	7.55	11.09	7.48	3.91	4.44	<0.001
**Visuospatial skills**							
ROCFT copy	26.18	7.94	27.43	6.94	20.60	9.59	<0.001
Clock drawing test	12.98	3.36	13.50	3.12	10.72	3.43	<0.001

*n* = 570 for full sample; *n* = 457 for participants without dementia (includes Subjective Cognitive Decline and amnestic Mild Cognitive Impairment); *n* = 113 for participants with dementia. All neuropsychological tests in the table are coded such that higher scores indicate better performance, except Trail Making Test Parts A and B.

AVLT 1–5, Rey Auditory Verbal Learning Test – the sum of scores from trials 1–5; AVLT 30, Rey Auditory Verbal Learning Test – delayed recall after 30 min; BNT, Boston Naming Test; BVMTR, Brief Visuospatial Memory Test – Revised; COWAT, Controlled Oral Word Association Test, letters N, K, and P; GDS-15, Geriatric Depression Scale-15 item version; Logical Memory, Uniform Data Set Logical Memory Test; M, mean; ROCFT, Rey–Osterrieth Complex Figure Test; SD, standard deviation; VFT, Verbal Fluency Test; WAIS-III, Wechsler Adult Intelligence Scale – III.

^a^Occupational position categorized according to the 2008 International Standard Classification of Occupations and reverse coded such that higher scores indicate higher occupational positions.

^b^Trail Making test Parts A and B: raw scores expressed in seconds to completion in table; for analyses, these scores were reverse coded.

^c^Assessed as subscores (number of correct items).

^d^Differences in variables between participants without and with dementia were assessed with *t*-tests or chi-square tests, where applicable.

### Participants without dementia

**FIGURE 1:**
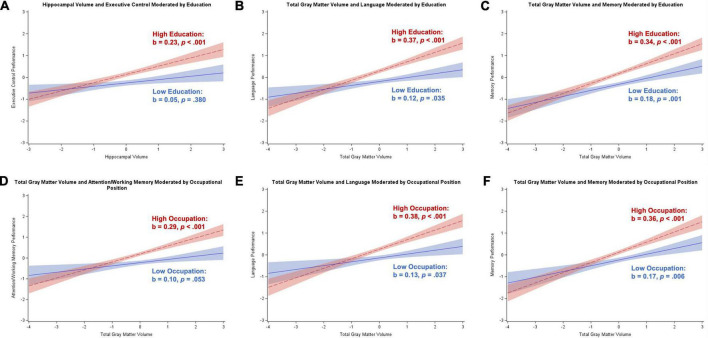
Plots of significant moderations by education and occupational position of the associations between cognition and brain volume in participants without dementia. High education or occupational position corresponds to 1 SD above the mean and low education or occupational position corresponds to 1 SD below the mean. The red dotted line represents high cognitive reserve, and the blue solid line represents low cognitive reserve. Shaded areas represent 95% confidence intervals. Cognition and brain volume were standardized. **(A)** Significant positive association between hippocampal volume and executive control for individuals with high education; non-significant for low education. **(B)** Significant positive association between total gray matter volume and language for individuals with low and high education; stronger relationship for high education. **(C)** Significant positive association between total gray matter volume and memory for individuals with low and high education; stronger relationship for high education. **(D)** Significant positive association between total gray matter volume and attention/working memory for individuals with high occupational position; non-significant for low occupational position. **(E)** Significant positive association between total gray matter volume and language for individuals with low and high occupational position; stronger relationship for high occupational position. **(F)** Significant positive association between total gray matter volume and memory for individuals with low and high occupational position; stronger relationship for high occupational position.

Table 2 contains results from the regression analyses for participants without dementia (i.e., SCD or aMCI), controlling for age, sex, and depressive symptoms. Results remained the same after dropping depressive symptoms as a covariate (results not shown). Hippocampal volume was positively related to executive control, language, memory, and visuospatial skills (*p*s < 0.001 to *p* = 0.006), whereas total gray matter volume was positively related to all five cognitive domains (all *p*s < 0.001). Education moderated the association between hippocampal volume and executive control (*b* = 0.09, *SE* = 0.04, *p* = 0.033), total gray matter volume and language (*b* = 0.12, *SE* = 0.04, *p* < 0.001), and total gray matter volume and memory (*b* = 0.08, *SE* = 0.03, *p* = 0.018). Application of the Johnson–Neyman technique revealed overall similar patterns of moderation by education for the hippocampal volume-executive control relationship and the relationships between total gray matter volume and language and memory. Specifically, there was a significant positive association between brain volume and these cognitive domains that corresponded to about 10–13 years of education, with the brain volume-cognition link getting stronger as education increased. Examination of Figures 1A–C illustrates a generally strong positive association between brain volume and cognition for high education, and either a weaker or non-significant positive association between brain volume and cognition for low education. Only the interaction between total gray matter volume and education in association with language remained significant after correction for multiple comparisons.

**TABLE 2:** Moderation analyses for participants without dementia.

	Hippocampal volume analyses						Total gray matter volume analyses					
		
	Years of education			Occupational position			Years of education			Occupational position		
				
Domain	*b*	*SE*	*p*	*b*	*SE*	*p*	*b*	*SE*	*p*	*b*	*SE*	*p*
**Attention/Working memory**												
Brain volume	0.06	0.04	0.150	0.07	0.04	0.072	**0.18**	**0.04**	<0.001*	**0.18**	**0.04**	<0.001*
CR proxy	**0.19**	**0.03**	<0.001*	**0.19**	**0.03**	<0.001*	**0.20**	**0.03**	<0.001*	**0.19**	**0.03**	<0.001*
Brain volume × CR proxy	0.06	0.03	0.077	0.06	0.04	0.154	0.04	0.03	0.245	**0.09**	**0.04**	**0.009^N.S.^**
**Executive control**												
Brain volume	**0.13**	**0.05**	0.006*	**0.15**	**0.05**	0.002*	**0.26**	**0.05**	<0.001*	**0.27**	**0.05**	<0.001*
CR proxy	**0.22**	**0.04**	<0.001*	**0.19**	**0.04**	<0.001*	**0.24**	**0.04**	<0.001*	**0.21**	**0.04**	<0.001*
Brain volume × CR proxy	**0.09**	**0.04**	**0.033^N.S.^**	0.06	0.05	0.180	0.06	0.04	0.121	0.04	0.04	0.321
**Language**												
Brain volume	**0.19**	**0.05**	<0.001*	**0.20**	**0.05**	<0.001*	**0.23**	**0.04**	<0.001*	**0.24**	**0.05**	<0.001*
CR proxy	**0.18**	**0.04**	<0.001*	**0.19**	**0.04**	<0.001*	**0.21**	**0.04**	<0.001*	**0.19**	**0.04**	<0.001*
Brain volume × CR proxy	0.07	0.04	0.058	0.06	0.04	0.191	**0.12**	**0.04**	<0.001*	**0.13**	**0.04**	0.002*
**Memory**												
Brain volume	**0.42**	**0.04**	<0.001*	**0.43**	**0.04**	<0.001*	**0.25**	**0.04**	<0.001*	**0.25**	**0.04**	<0.001*
CR proxy	**0.20**	**0.03**	<0.001*	**0.18**	**0.03**	<0.001*	**0.23**	**0.04**	<0.001*	**0.17**	**0.04**	<0.001*
Brain volume × CR proxy	0.04	0.03	0.286	0.05	0.04	0.153	**0.08**	**0.03**	**0.018^N.S.^**	**0.10**	**0.04**	**0.013^N.S.^**
**Visuospatial skills**												
Brain volume	**0.20**	**0.05**	<0.001*	**0.21**	**0.05**	<0.001*	**0.25**	**0.05**	<0.001*	**0.27**	**0.05**	<0.001*
CR proxy	0.01	0.04	0.882	0.04	0.04	0.337	0.03	0.04	0.513	0.06	0.04	0.215
Brain volume × CR proxy	0.03	0.04	0.474	−0.04	0.05	0.419	0.06	0.04	0.182	−0.01	0.05	0.828

Participants without dementia include individuals diagnosed with Subjective Cognitive Decline or amnestic Mild Cognitive Impairment. Analyses are from fully adjusted models which included the main effect of brain volume, the main effect of the cognitive reserve proxy, and the interaction between brain volume and the cognitive reserve proxy, also controlling for age, sex, and depressive symptoms. Education, occupational position, and all cognitive domains were standardized. Hippocampal volume and total gray matter volume were adjusted for estimated total intracranial volume then standardized. Bolded values indicate significant effects. b, unstandardized regression coefficient; CR, cognitive reserve; SE, standard error.

*Significant after Holm–Bonferroni correction.

^N.S.^Not significant after Holm–Bonferroni correction.

Occupational position moderated the association between total gray matter volume and attention/working memory (*b* = 0.09, *SE* = 0.04, *p* = 0.009), language (*b* = 0.13, *SE* = 0.04, *p* = 0.002), and memory (*b* = 0.10, *SE* = 0.04, *p* = 0.013). The Johnson–Neyman technique revealed similar moderating effects of occupational position in each cognitive domain. Specifically, there were significant positive associations between brain volume and cognition for individuals with over level 3 occupational positions (starting with skilled workers/service or sales workers), with the brain volume-cognition relationships strengthening as occupational position increased. Figures 1D–F illustrates a strong positive association between total gray matter volume and cognition for participants with high occupational positions and either a weaker or non-significant positive association for participants with low occupational positions. Similar to the education analyses, only the total gray matter volume-occupational position interaction relating to language remained significant after correcting for multiple comparisons.

#### Covariate effects for participants without dementia

Table 3 contains the effects of age, sex, and depressive symptoms from the moderation analyses conducted for participants without dementia. Across the two cognitive reserve proxies and two brain areas of interest, age was negatively related to all five cognitive outcomes for all but two models. Women performed better on executive control and worse on language and visuospatial skills compared to men. Having more depressive symptoms was related to worse attention/working memory, executive control, and visuospatial skills.

**TABLE 3:** Covariate effects from moderation analyses for participants without dementia.

	Hippocampal volume analyses						Total gray matter volume analyses					
		
	Years of education			Occupational position			Years of education			Occupational position		
				
Domain	*b*	*SE*	*p*	*b*	*SE*	*p*	*b*	*SE*	*p*	*b*	*SE*	*p*
**Attention/Working memory**												
Age	**−0.03**	**0.00**	**<0.001**	**−0.03**	**0.00**	**<0.001**	**−0.02**	**0.00**	**<0.001**	**−0.02**	**0.00**	**<0.001**
Sex	0.07	0.07	0.298	−0.03	0.07	0.607	0.03	0.07	0.649	−0.09	0.07	0.154
Depressive symptoms	**−0.04**	**0.01**	**<0.001**	**−0.04**	**0.01**	**<0.001**	**−0.04**	**0.01**	**<0.001**	**−0.04**	**0.01**	**<0.001**
**Executive control**												
Age	**−0.03**	**0.01**	**<0.001**	**−0.03**	**0.01**	**<0.001**	**−0.02**	**0.01**	**<0.001**	**−0.02**	**0.01**	**<0.001**
Sex	**0.25**	**0.09**	**0.003**	0.13	0.08	0.111	**0.20**	**0.08**	**0.018**	0.06	0.08	0.452
Depressive symptoms	**−0.04**	**0.01**	**0.007**	**−0.03**	**0.01**	**0.014**	**−0.03**	**0.01**	**0.017**	**−0.03**	**0.01**	**0.034**
**Language**												
Age	**−0.02**	**0.01**	**<0.001**	**−0.02**	**0.01**	**<0.001**	**−0.02**	**0.01**	**0.001**	**−0.02**	**0.01**	**<0.001**
Sex	−0.02	0.08	0.762	−0.13	0.08	0.102	−0.06	0.08	0.427	**−0.18**	**0.08**	**0.016**
Depressive symptoms	−0.02	0.01	0.050	−0.02	0.01	0.073	−0.02	0.01	0.188	−0.01	0.01	0.413
**Memory**												
Age	**−0.01**	**0.00**	**0.003**	**−0.02**	**0.00**	**<0.001**	**−0.02**	**0.00**	**<0.001**	**−0.03**	**0.01**	**<0.001**
Sex	0.01	0.07	0.863	−0.11	0.07	0.093	0.02	0.08	0.788	−0.12	0.07	0.097
Depressive symptoms	−0.02	0.01	0.081	−0.02	0.01	0.120	0.00	0.01	0.989	0.00	0.01	0.724
**Visuospatial skills**												
Age	**−0.02**	**0.01**	**0.010**	**−0.02**	**0.01**	**0.010**	−0.01	0.01	0.061	−0.01	0.01	0.054
Sex	**−0.24**	**0.09**	**0.012**	**−0.22**	**0.09**	**0.010**	**−0.28**	**0.09**	**0.003**	**−0.28**	**0.09**	**0.001**
Depressive symptoms	**−0.03**	**0.01**	**0.041**	**−0.03**	**0.01**	**0.035**	−0.02	0.01	0.151	−0.02	0.01	0.163

Analyses are from fully adjusted models which included the main effect of brain volume, the main effect of the cognitive reserve proxy, and the interaction between brain volume and the cognitive reserve proxy, also controlling for age, sex, and depressive symptoms. Age was measured in years, sex was measured with a binary variable (0 = male, 1 = female), and depressive symptoms were measured with the Geriatric Depression Scale, 15-item version, with higher scores indicating more depressive symptoms. Bolded values indicate significant effects. b, unstandardized regression coefficient; SE, standard error.

### Participants with dementia

**FIGURE 2:**
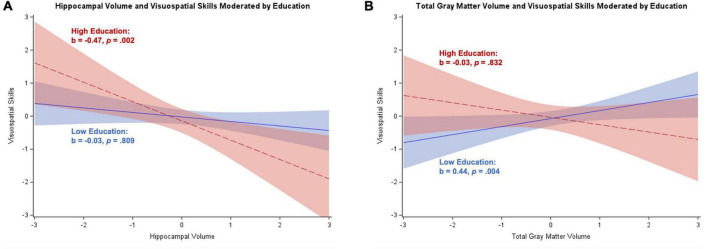
Plots of significant moderations by education of the associations between brain volume and visuospatial skills for participants with dementia. High education corresponds to 1 SD above the mean and low education corresponds to 1 SD below the mean. The red dotted line represents high education, and the blue solid line represents low education. Shaded areas represent 95% confidence intervals. Visuospatial skills and brain volume were standardized. **(A)** Significant negative relationship between hippocampal volume and visuospatial skills for individuals with high education; non-significant for low education. **(B)** Significant positive association between total gray matter volume and visuospatial skills for participants with low education; non-significant negative association for individuals with high education.

Table 4 contains results from the regression analyses for participants with dementia syndrome, controlling for age, sex, and depressive symptoms. Results after excluding depressive symptoms as a covariate were consistent with main analyses (results not shown). Hippocampal volume was negatively associated with attention/working memory and visuospatial skills, and positively related to memory (*p*s = 0.003–0.045). Total gray matter volume was positively related to executive control (*p* = 0.044). Education moderated the association between hippocampal volume and visuospatial skills (*b* = –0.26, *SE* = 0.11, *p* = 0.024), with the Johnson–Neyman technique identifying 12+ years of education associated with a significant negative effect. Figure 2A illustrates a strong negative relationship for participants with high education and a non-significant negative relationship for participants with low education. Education also moderated the association between total gray matter volume and visuospatial skills (*b* = –0.28, *SE* = 0.12, *p* = 0.024), but in this case, there was a significant positive relationship for participants with low-to-middle educational attainment (i.e., 8–13 years). Figure 2B illustrates a significant positive association between total gray matter volume and visuospatial skills for individuals with low education, and a non-significant negative relationship between these variables for individuals with high education. Correction for multiple comparisons reduced these two interaction effects to null.

**TABLE 4:** Moderation analyses for participants with dementia.

	Hippocampal volume analyses						Total gray matter volume analyses					
		
	Years of education			Occupational position			Years of education			Occupational position		
				
Domain	*b*	*SE*	*p*	*b*	*SE*	*p*	*b*	*SE*	*p*	*b*	*SE*	*p*
**Attention/Working memory**												
Brain volume	**−0.26**	**0.09**	**0.008^N.S.^**	**−0.18**	**0.09**	**0.036^N.S.^**	0.06	0.09	0.483	0.06	0.08	0.495
CR proxy	0.04	0.09	0.633	0.02	0.07	0.800	0.06	0.09	0.518	0.02	0.07	0.793
Brain volume × CR proxy	−0.13	0.09	0.150	−0.01	0.07	0.892	0.01	0.09	0.872	0.07	0.08	0.383
**Executive control**												
Brain volume	−0.24	0.13	0.072	−0.11	0.12	0.372	0.23	0.12	0.062	**0.23**	**0.11**	**0.044^N.S.^**
CR proxy	0.11	0.13	0.409	0.00	0.10	0.977	0.16	0.13	0.231	0.03	0.10	0.737
Brain volume × CR proxy	−0.20	0.12	0.115	0.02	0.09	0.799	−0.04	0.12	0.741	0.06	0.10	0.542
**Language**												
Brain volume	−0.09	0.11	0.413	−0.05	0.10	0.582	0.06	0.10	0.556	0.10	0.09	0.298
CR proxy	−0.02	0.10	0.836	0.08	0.08	0.301	−0.00	0.11	0.992	0.10	0.08	0.219
Brain volume × CR proxy	−0.11	0.10	0.288	−0.06	0.07	0.442	−0.05	0.10	0.655	−0.02	0.09	0.851
**Memory**												
Brain volume	**0.16**	**0.08**	**0.045^N.S.^**	**0.17**	**0.07**	**0.022^N.S.^**	0.04	0.08	0.606	0.01	0.07	0.833
CR proxy	0.07	0.08	0.330	0.04	0.06	0.480	0.06	0.08	0.464	0.04	0.06	0.529
Brain volume × CR proxy	0.06	0.08	0.467	0.08	0.05	0.164	0.08	0.08	0.317	0.10	0.06	0.139
**Visuospatial skills**												
Brain volume	**−0.37**	**0.12**	0.003*	−0.17	0.11	0.111	0.08	0.11	0.486	0.19	0.11	0.072
CR proxy	−0.13	0.11	0.260	−0.08	0.09	0.375	−0.05	0.12	0.644	−0.06	0.09	0.533
Brain volume × CR proxy	**−0.26**	**0.11**	**0.024^N.S.^**	0.03	0.08	0.676	**−0.28**	**0.12**	**0.024^N.S.^**	−0.01	0.10	0.917

Analyses are from fully adjusted models which included the main effect of brain volume, the main effect of the cognitive reserve proxy, and the interaction between brain volume and the cognitive reserve proxy, also controlling for age, sex, and depressive symptoms. Education, occupational position, and all cognitive domains were standardized. Hippocampal volume and total gray matter volume were adjusted for estimated total intracranial volume then standardized. Bolded values indicate significant effects. b, unstandardized regression coefficient; CR, cognitive reserve; SE, standard error.

*Significant after Holm–Bonferroni correction.

^N.S.^Not significant after Holm–Bonferroni correction.

In sensitivity analyses, we assessed whether effects were similar when the sample was restricted to the 84 participants who had an AD etiology (pure or mixed). Hippocampal volume was negatively related to visuospatial skills (*b* = –0.29, *SE* = 0.15, *p* = 0.049), total gray matter volume was positively associated with executive control performance (analyses with education as moderator: *b* = 0.30, *SE* = 0.13, *p* = 0.027; analyses with occupational position as moderator: *b* = 0.29, *SE* = 0.12, *p* = 0.022), and education was positively related to executive control performance (total gray matter volume analyses: *b* = 0.29, *SE* = 0.15, *p* = 0.049). No other main effects were significant and there was no evidence that education or occupational position moderated the association between brain volume and cognition.

#### Covariate effects for participants with dementia

Table 5 contains the effects of age, sex, and depressive symptoms from the moderation analyses conducted for participants with dementia. Age was negatively related to memory performance. There were no main effects of sex in any of the models conducted. Having more depressive symptoms was related to better memory performance but worse executive control and visuospatial skills.

**TABLE 5:** Covariate effects from moderation analyses for participants with dementia.

	Hippocampal volume analyses						Total gray matter volume analyses					
		
	Years of education			Occupational position			Years of education			Occupational position		
				
Domain	*b*	*SE*	*p*	*b*	*SE*	*p*	*b*	*SE*	*p*	*b*	*SE*	*p*
**Attention/Working memory**												
Age	−0.01	0.01	0.324	−0.01	0.01	0.360	−0.00	0.01	0.955	−0.01	0.01	0.614
Sex	0.12	0.16	0.474	0.06	0.16	0.679	0.00	0.17	0.977	−0.03	0.16	0.855
Depressive symptoms	−0.02	0.02	0.275	−0.02	0.02	0.325	−0.03	0.02	0.185	−0.03	0.02	0.153
**Executive control**												
Age	0.00	0.01	0.733	0.00	0.01	0.823	0.02	0.01	0.143	0.01	0.01	0.380
Sex	0.38	0.23	0.104	0.26	0.22	0.235	0.21	0.23	0.360	0.11	0.22	0.624
Depressive symptoms	−0.05	0.03	0.092	−0.05	0.03	0.126	−0.06	0.03	0.055	**−0.06**	**0.03**	**0.046**
**Language**												
Age	−0.01	0.01	0.476	−0.01	0.01	0.543	−0.00	0.01	0.816	−0.00	0.01	0.717
Sex	−0.19	0.19	0.308	−0.20	0.18	0.257	−0.24	0.19	0.214	−0.26	0.18	0.146
Depressive symptoms	0.01	0.02	0.623	0.02	0.02	0.512	0.01	0.02	0.692	0.01	0.02	0.627
**Memory**												
Age	**−0.02**	**0.01**	**0.047**	**−0.02**	**0.01**	**0.011**	**−0.02**	**0.01**	**0.015**	**−0.03**	**0.01**	**0.003**
Sex	−0.10	0.14	0.473	−0.14	0.13	0.283	−0.07	0.14	0.609	−0.09	0.13	0.484
Depressive symptoms	0.03	0.02	0.064	0.03	0.02	0.077	**0.04**	**0.02**	**0.029**	**0.04**	**0.02**	**0.043**
**Visuospatial skills**												
Age	−0.00	0.01	0.933	0.00	0.01	0.843	0.02	0.01	0.163	0.02	0.01	0.200
Sex	−0.12	0.21	0.561	−0.11	0.20	0.576	−0.25	0.21	0.241	−0.28	0.20	0.168
Depressive symptoms	**−0.07**	**0.03**	**0.010**	**−0.06**	**0.03**	**0.037**	**−0.09**	**0.03**	**0.002**	**−0.07**	**0.03**	**0.009**

Analyses are from fully adjusted models which included the main effect of brain volume, the main effect of the cognitive reserve proxy, and the interaction between brain volume and the cognitive reserve proxy, also controlling for age, sex, and depressive symptoms. Age was measured in years, sex was measured with a binary variable (0 = male, 1 = female), and depressive symptoms were measured with the Geriatric Depression Scale, 15-item version, with higher scores indicating more depressive symptoms. Bolded values indicate significant effects. b, unstandardized regression coefficient; SE, standard error.

### Supplemental analyses

#### Moderation analyses separating participants without dementia

In order to provide a more refined tracking of how cognitive reserve may modify the relationship between brain volume and cognitive outcomes across the cognitive status continuum, we ran supplemental analyses for participants without dementia separately for those with SCD and aMCI.

##### Participants with subjective cognitive decline

**FIGURE 3:**
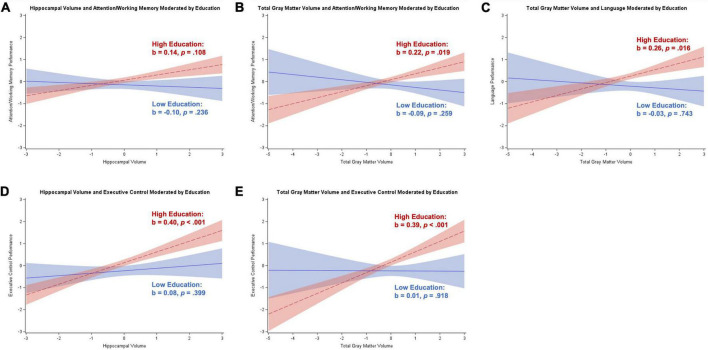
Plots of significant moderations by education of the associations between brain volume and cognition for participants with subjective cognitive decline. High education corresponds to 1 SD above the mean and low education corresponds to 1 SD below the mean. The red dotted line represents high education, and the blue solid line represents low education. Shaded areas represent 95% confidence intervals. Cognition and brain volume were standardized. **(A)** Non-significant positive association between hippocampal volume and attention/working memory for participants with high education; non-significant negative association for low education. **(B)** Significant positive association between total gray matter volume and attention/working memory for participants with high education; non-significant negative association for low education. **(C)** Significant positive association between total gray matter volume and language for participants with high education; non-significant negative association for low education. **(D)** Significant positive association between hippocampal volume and executive control for participants with high education; non-significant positive association for low education. **(E)** Significant positive association between total gray matter volume and executive control for participants with high education; non-significant positive association for low education.

Moderation analyses for participants with SCD revealed a positive relationship between hippocampal volume and executive control, memory, and visuospatial skills (*p*s < 0.001 to *p* = 0.045), and a positive relationship between total gray matter volume and visuospatial skills (*p* < 0.001). Education moderated the relationship between hippocampal volume and attention/working memory (*b* = 0.12, *SE* = 0.05, *p* = 0.020) and executive control (*b* = 0.17, *SE* = 0.06, *p* = 0.009), and total gray matter volume and attention/working memory (*b* = 0.16, *SE* = 0.06, *p* = 0.010), executive control (*b* = 0.20, *SE* = 0.08, *p* = 0.012), and language (*b* = 0.15, *SE* = 0.07, *p* = 0.037). The Johnson–Neyman technique revealed a significant positive relationship between brain volume and cognition starting between 14 and 21 years of education that strengthened as education increased. There was also a significant negative relationship between total gray matter volume and attention/working memory for participants with less than 9 years of education that strengthened with decreasing education. These results are illustrated in Figure 3.

**FIGURE 4:**
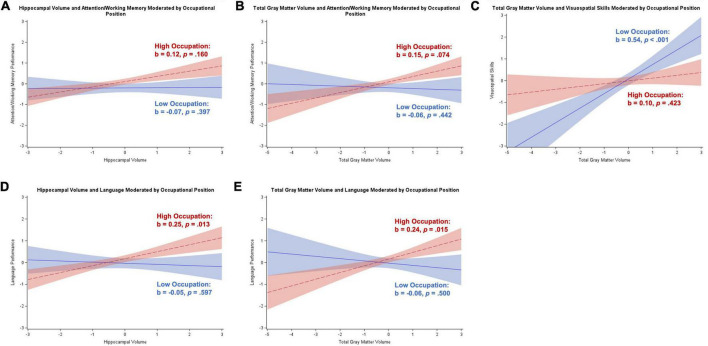
Plots of significant moderations by occupational position of the associations between brain volume and cognition for participants with subjective cognitive decline. High occupational position corresponds to 1 SD above the mean and low occupational position corresponds to 1 SD below the mean. The red dotted line represents high occupational position, and the blue solid line represents low occupational position. Shaded areas represent 95% confidence intervals. Cognition and brain volume were standardized. **(A)** Non-significant positive association between hippocampal volume and attention/working memory for participants with high occupational position; non-significant negative association for low occupational position. **(B)** Non-significant positive association between total gray matter volume and attention/working memory for participants with high occupational position; non-significant negative association for low occupational position. **(C)** Non-significant positive association between total gray matter volume and visuospatial skills for participants with high occupational position; significant positive association for low occupational position. **(D)** Significant positive association between hippocampal volume and language for participants with high occupational position; non-significant negative association for low occupational position. **(E)** Significant positive association between total gray matter volume and language for participants with high occupational position; non-significant negative association for low occupational position.

Occupational position moderated the relationship between hippocampal volume and attention/working memory (*b* = 0.11, *SE* = 0.06, *p* = 0.046) and language (*b* = 0.18, *SE* = 0.07, *p* = 0.007), and total gray matter volume and attention/working memory (*b* = 0.13, *SE* = 0.06, *p* = 0.038), language (*b* = 0.19, *SE* = 0.07, *p* = 0.012), and visuospatial skills (*b* = –0.27, *SE* = 0.09, *p* = 0.004). The Johnson–Neyman technique revealed no regions of significance for the relationships between brain volume and attention/working memory, implying a more general trend as opposed to a specific region of significance. For language, there were significant negative relationships with brain volume for participants with either “plant and machine operators and assemblers” occupations or “elementary” occupations (≤1 on scale), but also significant positive relationships with brain volume for participants with managerial or professional occupations (≥7 on scale). For visuospatial skills, participants with less than professional occupations (<7 on scale) had a significant positive association with total gray matter volume that strengthened with decreasing occupational position. These results are illustrated in Figure 4.

##### Participants with amnestic mild cognitive impairment

**FIGURE 5:**
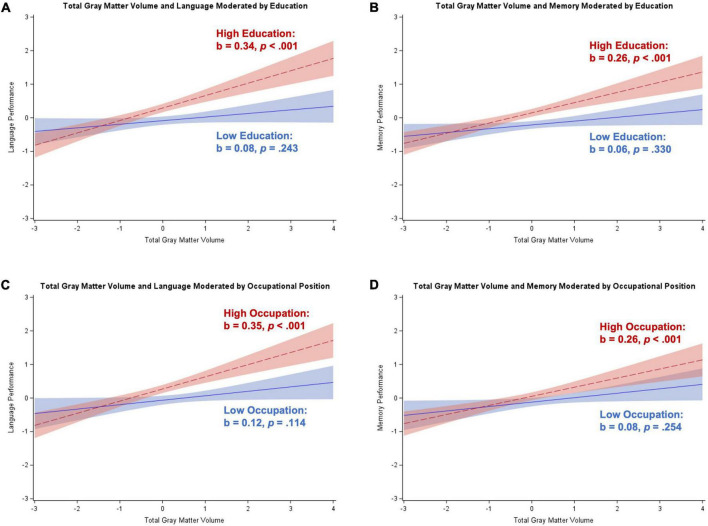
Plots of significant moderations by education and occupational position of the associations between total gray matter volume and cognition for participants with amnestic mild cognitive impairment. High education or occupational position corresponds to 1 SD above the mean and low education or occupational position corresponds to 1 SD below the mean. The red dotted line represents high education or occupational position, and the blue solid line represents low education or occupational position. Shaded areas represent 95% confidence intervals. Cognition and total gray matter volume were standardized. **(A)** Significant positive association between total gray matter volume and language for participants with high education; non-significant positive association for low education. **(B)** Significant positive association between total gray matter volume and memory for participants with high education; non-significant positive association for low education. **(C)** Significant positive association between total gray matter volume and language for participants with high occupational position; non-significant positive association for low occupational position. **(D)** Significant positive association between total gray matter volume and memory for participants with high occupational position; non-significant positive association for low occupational position.

Moderation analyses for participants with aMCI revealed a significant positive association between hippocampal volume and memory (*p* < 0.001) and significant positive associations between total gray matter volume and each of the five cognitive domains (*p*s < 0.001 to *p* = 0.018). Education moderated the association between total gray matter volume and language (*b* = 0.13, *SE* = 0.04, *p* = 0.003) and memory (*b* = 0.10, *SE* = 0.04, *p* = 0.011). The Johnson–Neyman technique revealed that for both language and memory, there was a significant positive association with total gray matter volume for participants with over 12 years of education that strengthened with increasing education (see Figures 5A,B).

Occupational position also moderated the association between total gray matter volume and language (*b* = 0.12, *SE* = 0.05, *p* = 0.014) and memory (*b* = 0.09, *SE* = 0.04, *p* = 0.039). The Johnson–Neyman technique revealed that there was a significant positive association between total gray matter volume and both language and memory for participants with at least “services and sales workers” occupations (>4 on scale) that strengthened with increasing occupational position (see Figures 5C,D).

#### Restricting to participants with data on main lifetime occupation

Restricting our occupational position analyses to participants with data available for main lifetime occupation reduced our sample to 373 participants (311 without dementia; 62 with dementia). For participants without dementia, similar to main analyses, occupational position was positively related to attention/working memory, executive control, language, and memory performance (all *p*s < 0.001). However, whereas in main analyses occupational position moderated the relationship between total gray matter volume and attention/working memory, language, and memory, analyses restricted to participants with data on main lifetime occupation revealed occupational position moderated the association between hippocampal volume and attention/working memory (*b* = 0.10, *SE* = 0.05, *p* = 0.031) and memory (*b* = 0.11, *SE* = 0.05, *p* = 0.018). Similar to main analyses, higher occupational position magnified the association between brain volume and cognition, such that having a higher occupational position was related to a stronger positive relationship between hippocampal volume and attention/working memory and memory. Thus, in sum, the moderating effect was still present in two of the three original cognitive domains, although it was present for a different brain region.

For participants with dementia, using main lifetime occupation as the proxy (rather than information about main lifetime occupation supplemented with last occupation when main occupation was not available) revealed an interaction between hippocampal volume and occupational position on language performance (*b* = –0.28, *SE* = 0.11, *p* = 0.016). The Johnson–Neyman technique indicated that higher occupational position was related to a stronger negative association between hippocampal volume and language performance. This is similar to effects found in main analyses although for a different cognitive domain.

#### Assessing the influence of sex on reserve/resilience effects

The main analyses were also conducted separated for men and women in order to observe whether sex influenced the moderating effect of cognitive reserve proxies on the association between brain volume and cognition. Like main analyses, these models were conducted separately for participants without and with dementia. Fully adjusted models controlled for age and depressive symptoms. Analyses for men included 197 participants without dementia and 41 participants with dementia. Analyses for women included 259 participants without dementia and 72 participants with dementia. Table 6 presents results for men and women without dementia and Table 7 presents results for men and women with dementia.

**TABLE 6:** Sensitivity moderation analyses for participants without dementia stratified by sex.

	Hippocampal volume analyses						Total gray matter volume analyses					
		
	Years of education			Occupational position			Years of education			Occupational position		
				
Domain	*b*	*SE*	*p*	*b*	*SE*	*p*	*b*	*SE*	*p*	*b*	*SE*	*p*

	*Analyses for men*											
**Attention/Working memory**												
Brain volume	0.01	0.07	0.832	0.07	0.06	0.253	**0.16**	**0.06**	**0.011**	**0.18**	**0.06**	**0.002**
CR proxy	**0.24**	**0.05**	**<0.001**	**0.22**	**0.05**	**<0.001**	**0.25**	**0.05**	**<0.001**	**0.23**	**0.04**	**<0.001**
Brain volume × CR proxy	0.10	0.06	0.078	0.08	0.06	0.140	0.07	0.05	0.148	**0.11**	**0.05**	**0.022**
**Executive control**												
Brain volume	0.10	0.09	0.226	**0.18**	**0.08**	**0.029**	**0.32**	**0.07**	**<0.001**	**0.37**	**0.07**	**<0.001**
CR proxy	**0.26**	**0.06**	**<0.001**	**0.15**	**0.06**	**0.009**	**0.27**	**0.06**	**<0.001**	**0.16**	**0.05**	**0.004**
Brain volume × CR proxy	0.10	0.07	0.157	0.02	0.07	0.795	0.04	0.06	0.438	−0.05	0.06	0.444
**Language**												
Brain volume	0.14	0.07	0.065	**0.21**	**0.07**	**0.003**	**0.24**	**0.06**	**<0.001**	**0.30**	**0.06**	**<0.001**
CR proxy	**0.14**	**0.06**	**0.017**	**0.15**	**0.05**	**0.002**	**0.18**	**0.06**	**0.002**	**0.16**	**0.05**	**<0.001**
Brain volume × CR proxy	0.10	0.06	0.126	0.02	0.06	0.697	**0.12**	**0.05**	**0.016**	0.07	0.05	0.179
**Memory**												
Brain volume	**0.38**	**0.06**	**<0.001**	**0.41**	**0.06**	**<0.001**	**0.22**	**0.06**	**<0.001**	**0.26**	**0.06**	**<0.001**
CR proxy	**0.16**	**0.05**	**0.001**	**0.14**	**0.04**	**<0.001**	**0.20**	**0.05**	**<0.001**	**0.13**	**0.05**	**0.003**
Brain volume × CR proxy	0.05	0.05	0.325	0.06	0.05	0.217	**0.10**	**0.05**	**0.049**	0.06	0.05	0.207
**Visuospatial skills**												
Brain volume	0.08	0.08	0.318	0.14	0.07	0.057	0.07	0.07	0.324	0.12	0.07	0.094
CR proxy	0.06	0.06	0.347	0.05	0.05	0.300	0.09	0.06	0.148	0.06	0.05	0.261
Brain volume × CR proxy	0.10	0.07	0.127	0.01	0.06	0.878	**0.12**	**0.06**	**0.032**	0.06	0.06	0.263

	***Analyses for women***											

**Attention/Working memory**												
Brain volume	0.07	0.05	0.163	0.07	0.05	0.180	**0.19**	**0.05**	**<0.001**	**0.19**	**0.05**	**<0.001**
CR proxy	**0.16**	**0.05**	**0.002**	**0.15**	**0.05**	**0.008**	**0.17**	**0.05**	**<0.001**	**0.14**	**0.06**	**0.013**
Brain volume × CR proxy	0.06	0.05	0.263	0.05	0.06	0.446	0.04	0.06	0.486	0.11	0.06	0.079
**Executive control**												
Brain volume	**0.17**	**0.06**	**0.008**	**0.14**	**0.06**	**0.019**	**0.25**	**0.06**	**<0.001**	**0.23**	**0.06**	**<0.001**
CR proxy	**0.15**	**0.06**	**0.011**	**0.22**	**0.06**	**<0.001**	**0.17**	**0.06**	**0.005**	**0.21**	**0.07**	**0.002**
Brain volume × CR proxy	**0.14**	**0.06**	**0.026**	0.10	0.07	0.158	0.13	0.07	0.057	**0.15**	**0.07**	**0.039**
**Language**												
Brain volume	**0.22**	**0.06**	**<0.001**	**0.21**	**0.06**	**<0.001**	**0.25**	**0.07**	**<0.001**	**0.22**	**0.06**	**<0.001**
CR proxy	**0.22**	**0.06**	**<0.001**	**0.20**	**0.07**	**0.002**	**0.22**	**0.06**	**<0.001**	**0.17**	**0.07**	**0.012**
Brain volume × CR proxy	0.07	0.06	0.286	0.09	0.07	0.206	**0.14**	**0.07**	**0.038**	**0.21**	**0.07**	**0.005**
**Memory**												
Brain volume	**0.45**	**0.05**	**<0.001**	**0.45**	**0.05**	**<0.001**	**0.30**	**0.06**	**<0.001**	**0.28**	**0.06**	**<0.001**
CR proxy	**0.22**	**0.05**	**<0.001**	**0.20**	**0.06**	**<0.001**	**0.23**	**0.06**	**<0.001**	**0.17**	**0.06**	**0.008**
Brain volume × CR proxy	0.05	0.05	0.343	0.05	0.06	0.398	0.12	0.06	0.055	**0.17**	**0.07**	**0.014**
**Visuospatial skills**												
Brain volume	**0.26**	**0.07**	**<0.001**	**0.26**	**0.07**	**<0.001**	**0.40**	**0.08**	**<0.001**	**0.39**	**0.08**	**<0.001**
CR proxy	−0.02	0.07	0.780	0.04	0.08	0.580	−0.02	0.07	0.788	0.08	0.08	0.307
Brain volume × CR proxy	0.03	0.07	0.652	−0.08	0.08	0.324	0.12	0.08	0.137	−0.08	0.08	0.346

Analyses are from fully adjusted models which included the main effect of brain volume, the main effect of the cognitive reserve proxy, and the interaction between brain volume and the cognitive reserve proxy, also controlling for age and depressive symptoms. Education, occupational position, and all cognitive domains were standardized. Hippocampal volume and total gray matter volume were adjusted for estimated total intracranial volume then standardized. Bolded values indicate significant effects. b, unstandardized regression coefficient; CR, cognitive reserve; SE, standard error.

**TABLE 7:** Sensitivity moderation analyses for participants with dementia stratified by sex.

	Hippocampal volume analyses						Total gray matter volume analyses					
		
	Years of education			Occupational position			Years of education			Occupational position		
				
Domain	*b*	*SE*	*p*	*b*	*SE*	*p*	*b*	*SE*	*p*	*b*	*SE*	*p*

	***Analyses for men***											
**Attention/Working memory**												
Brain volume	−0.23	0.15	0.139	−0.28	0.17	0.101	0.04	0.13	0.759	0.06	0.13	0.638
CR proxy	0.03	0.16	0.855	−0.01	0.11	0.956	0.02	0.15	0.891	0.04	0.12	0.728
Brain volume × CR proxy	0.17	0.19	0.387	−0.02	0.12	0.842	0.20	0.14	0.172	0.12	0.11	0.309
**Executive control**												
Brain volume	−0.24	0.20	0.239	−0.18	0.21	0.409	0.11	0.17	0.499	0.12	0.17	0.485
CR proxy	0.24	0.21	0.266	0.10	0.14	0.465	0.21	0.19	0.287	0.11	0.15	0.486
Brain volume × CR proxy	0.25	0.25	0.317	0.18	0.15	0.229	0.20	0.19	0.295	0.15	0.15	0.299
**Language**												
Brain volume	−0.03	0.18	0.866	−0.10	0.19	0.626	−0.05	0.15	0.758	−0.04	0.15	0.792
CR proxy	0.15	0.19	0.427	0.04	0.13	0.765	0.15	0.17	0.367	0.04	0.13	0.737
Brain volume × CR proxy	0.03	0.23	0.889	−0.07	0.13	0.600	0.09	0.16	0.591	−0.02	0.13	0.884
**Memory**												
Brain volume	0.02	0.11	0.889	0.06	0.12	0.616	0.05	0.09	0.621	0.02	0.09	0.788
CR proxy	0.03	0.12	0.828	−0.05	0.08	0.517	0.09	0.11	0.415	−0.01	0.08	0.948
Brain volume × CR proxy	−0.09	0.14	0.554	0.03	0.09	0.730	0.07	0.10	0.527	0.14	0.08	0.075
**Visuospatial skills**												
Brain volume	−0.35	0.17	0.052	−0.36	0.20	0.077	0.03	0.16	0.868	0.01	0.16	0.955
CR proxy	0.22	0.18	0.248	0.04	0.13	0.773	0.24	0.18	0.187	0.05	0.14	0.740
Brain volume × CR proxy	0.01	0.22	0.962	0.00	0.13	0.978	0.08	0.18	0.644	0.10	0.14	0.484

	***Analyses for women***											

**Attention/Working memory**												
Brain volume	**−0.42**	**0.13**	**0.003**	−0.15	0.10	0.162	−0.36	0.19	0.070	0.05	0.12	0.701
CR proxy	0.23	0.13	0.069	0.04	0.11	0.680	**0.37**	**0.15**	**0.019**	0.05	0.12	0.675
Brain volume × CR proxy	**−0.36**	**0.13**	**0.006**	−0.02	0.09	0.844	**−0.54**	**0.21**	**0.012**	−0.01	0.14	0.967
**Executive control**												
Brain volume	**−0.39**	**0.19**	**0.045**	−0.08	0.14	0.591	−0.05	0.27	0.855	0.29	0.16	0.084
CR proxy	0.35	0.20	0.094	−0.05	0.15	0.735	0.40	0.22	0.074	−0.03	0.17	0.843
Brain volume × CR proxy	**−0.42**	**0.19**	**0.027**	−0.02	0.12	0.898	−0.48	0.29	0.109	0.05	0.18	0.783
**Language**												
Brain volume	−0.07	0.16	0.666	−0.04	0.11	0.746	0.25	0.22	0.251	0.17	0.13	0.192
CR proxy	−0.09	0.15	0.549	0.11	0.12	0.331	−0.15	0.17	0.395	0.13	0.13	0.324
Brain volume × CR proxy	−0.05	0.15	0.751	−0.03	0.10	0.774	0.16	0.23	0.506	0.00	0.15	0.995
**Memory**												
Brain volume	**0.25**	**0.12**	**0.041**	**0.18**	**0.09**	**0.046**	−0.05	0.18	0.796	−0.04	0.10	0.723
CR proxy	0.07	0.11	0.525	0.11	0.09	0.220	0.11	0.14	0.441	0.15	0.11	0.161
Brain volume × CR proxy	0.13	0.11	0.251	0.07	0.08	0.350	−0.02	0.19	0.927	−0.06	0.12	0.614
**Visuospatial skills**												
Brain volume	−0.29	0.17	0.092	−0.09	0.13	0.483	−0.10	0.23	0.668	**0.32**	**0.15**	**0.031**
CR proxy	**−0.35**	**0.17**	**0.046**	−0.21	0.13	0.126	−0.13	0.18	0.474	−0.13	0.15	0.395
Brain volume × CR proxy	−0.17	0.17	0.305	0.11	0.11	0.336	**−0.55**	**0.26**	**0.037**	−0.00	0.17	0.988

Analyses are from fully adjusted models which included the main effect of brain volume, the main effect of the cognitive reserve proxy, and the interaction between brain volume and the cognitive reserve proxy, also controlling for age and depressive symptoms. Education, occupational position, and all cognitive domains were standardized. Hippocampal volume and total gray matter volume were adjusted for estimated total intracranial volume then standardized. Bolded values indicate significant effects. b, unstandardized regression coefficient; CR, cognitive reserve; SE, standard error.

##### Moderation analyses for men

**FIGURE 6:**
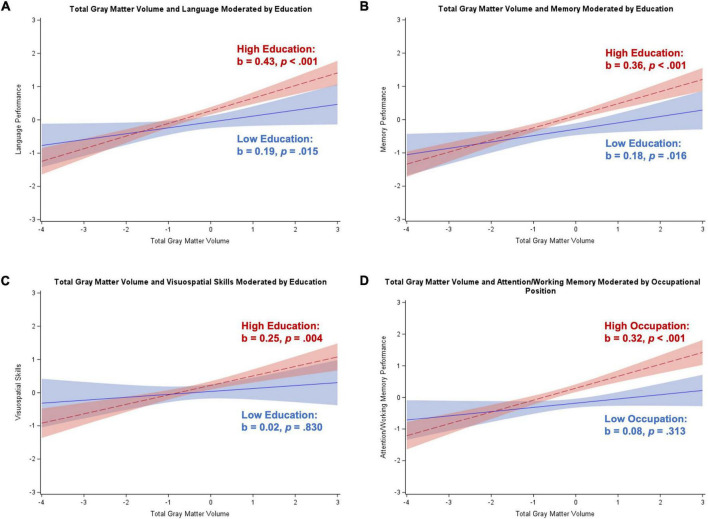
Plots of significant moderations by education and occupational position of the associations between total gray matter volume and cognition for men without dementia. High education or occupational position corresponds to 1 SD above the mean and low education or occupational position corresponds to 1 SD below the mean. The red dotted line represents high education or occupational position, and the blue solid line represents low education or occupational position. Shaded areas represent 95% confidence intervals. Cognition and total gray matter volume were standardized. **(A)** Significant positive association between total gray matter volume and language for participants with high education; significant positive association for low education. **(B)** Significant positive association between total gray matter volume and memory for participants with high education; significant positive association for low education. **(C)** Significant positive association between total gray matter volume and visuospatial skills for participants with high education; non-significant positive association for low education. **(D)** Significant positive association between total gray matter volume and attention/working memory for participants with high occupational position; non-significant positive association for low occupational position.

For men without dementia, hippocampal volume was positively related to executive control, language, and memory performance (*p*s < 0.001 to *p* = 0.029). Total gray matter volume was positively related to attention/working memory, executive control, language, and memory performance (*p*s < 0.001 to *p* = 0.011). Education moderated the association between total gray matter volume and language (*b* = 0.12, *SE* = 0.05, *p* = 0.016), memory (*b* = 0.10, *SE* = 0.05, *p* = 0.049), and visuospatial skills (*b* = 0.12, *SE* = 0.06, *p* = 0.032). The Johnson–Neyman technique revealed that there was a significant positive association between total gray matter volume and cognition starting between 12 and 16 years of education, with the association strengthening with increasing education. These results are illustrated in Figures 6A–C.

Occupational position moderated the association between total gray matter volume and attention/working memory in men without dementia (*b* = 0.11, *SE* = 0.05, *p* = 0.022). The Johnson–Neyman technique revealed that participants with around “clerical support workers” occupations (>4.7 on scale) had a significant positive association between total gray matter volume and attention/working memory that strengthened with increasing occupational position (see Figure 6D). There were no significant main effects or interactions for analyses in men with dementia.

##### Moderation analyses for women

**FIGURE 7:**
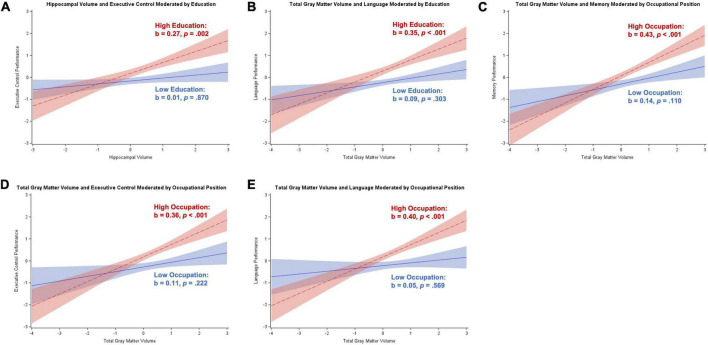
Plots of significant moderations by education and occupational position of the associations between brain volume and cognition for women without dementia. High education or occupational position corresponds to 1 SD above the mean and low education or occupational position corresponds to 1 SD below the mean. The red dotted line represents high education or occupational position, and the blue solid line represents low education or occupational position. Shaded areas represent 95% confidence intervals. Cognition and brain volume were standardized. **(A)** Significant positive association between hippocampal volume and executive control for participants with high education; non-significant positive association for low education. **(B)** Significant positive association between total gray matter volume and language for participants with high education; non-significant positive association for low education. **(C)** Significant positive association between total gray matter volume and memory for participants with high occupational position; non-significant positive association for low occupational position. **(D)** Significant positive association between total gray matter volume and executive control for participants with high occupational position; non-significant positive association for low occupational position. **(E)** Significant positive association between total gray matter volume and language for participants with high occupational position; non-significant positive association for low occupational position.

For women without dementia, hippocampal volume was positively related to executive control, language, memory, and visuospatial skills (*p*s < 0.001 to *p* = 0.019). Total gray matter volume was positively related to all five cognitive domains (all *p*s < 0.001). Education moderated the associations between hippocampal volume and executive control (*b* = 0.14, *SE* = 0.06, *p* = 0.026) and total gray matter volume and language (*b* = 0.14, *SE* = 0.07, *p* = 0.038). The Johnson–Neyman technique revealed that women with greater than 12–13 years of education had a positive association between brain volume and cognition that strengthened with increasing education (see Figures 7A,B).

Occupational position moderated the associations between total gray matter volume and executive control (*b* = 0.15, *SE* = 0.07, *p* = 0.039), language (*b* = 0.21, *SE* = 0.07, *p* = 0.005), and memory (*b* = 0.17, *SE* = 0.07, *p* = 0.014). The Johnson–Neyman technique revealed that women with between “services and sales workers” and “clerical support workers” occupations (4–5 on scale) had a significant positive association between total gray matter volume and cognition that strengthened with increasing occupational position (see Figures 7C–E).

**FIGURE 8:**
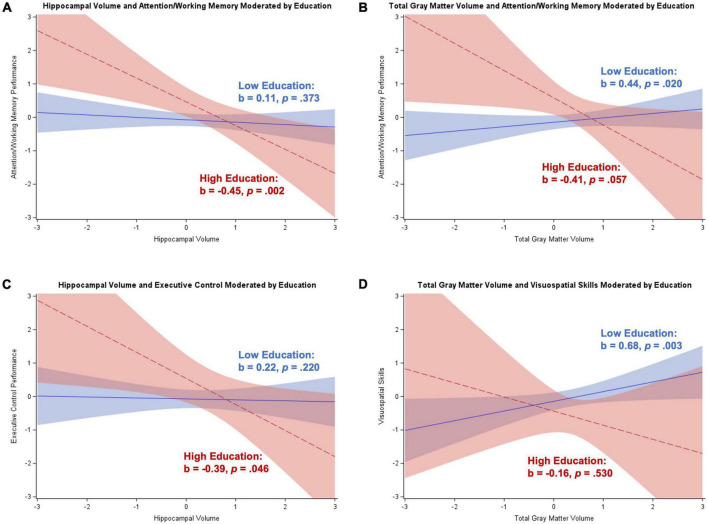
Plots of significant moderations by education of the associations between brain volume and cognition for women with dementia. High education corresponds to 1 SD above the mean and low education corresponds to 1 SD below the mean. The red dotted line represents high education, and the blue solid line represents low education. Shaded areas represent 95% confidence intervals. Cognition and brain volume were standardized. **(A)** Significant negative association between hippocampal volume and attention/working memory for participants with high education; non-significant positive association for low education. **(B)** Non-significant negative association between total gray matter volume and attention/working memory for participants with high education; significant positive association for low education. **(C)** Significant negative association between hippocampal volume and executive control for participants with high education; non-significant positive association for low education. **(D)** Non-significant negative association between total gray matter volume and visuospatial skills for participants with high education; significant positive association for low education.

For women with dementia, hippocampal volume was negatively related to attention/working memory and executive control and positively related to memory (*p* = 0.003 to *p* = 0.046). Total gray matter volume was positively related to visuospatial skills (*p* = 0.031). Education moderated the associations between hippocampal volume and attention/working memory (*b* = –0.36, *SE* = 0.13, *p* = 0.006) and executive control (*b* = –0.42, *SE* = 0.19, *p* = 0.027), and total gray matter volume and attention/working memory (*b* = –0.54, *SE* = 0.21, *p* = 0.012) and visuospatial skills (*b* = –0.55, *SE* = 0.26, *p* = 0.037). The Johnson–Neyman technique revealed that the relationship between total gray matter volume and attention/working memory and visuospatial skills was positive for women with less than 10–12 years of education that strengthened as education decreased. Additionally, for the brain volume and attention/working memory and executive control relationships, there was a significant negative association for women with between 12 and 15 years of education that strengthened with increasing education. These results are illustrated in Figure 8.

## Discussion

We examined the moderating effect of cognitive reserve on the association between brain volume and common cognitive domains in participants without dementia (i.e., SCD and aMCI) and with dementia syndrome. Our results indicate that the effect of cognitive reserve on the relationship between brain volume and cognition depends on (a) what diagnostic group is under investigation, (b) what cognitive domain is included, and (c) what is used as the proxy for cognitive reserve. For participants already experiencing cognitive difficulties but who are still free of dementia (i.e., SCD and aMCI), we found some evidence of a protective effect of both education and occupational position, whereby among those with higher cognitive reserve, brain volume and cognitive performance (i.e., attention/working memory, executive control, language, and memory) were more closely related than among those with low cognitive reserve. Therefore, those with high cognitive reserve appeared to be able to utilize available neural resources, when still available despite existing cognitive problems, more efficiently than those with low reserve. Caution is warranted in interpreting these interactions as only the association between total gray matter volume and language was significantly moderated by education after correction for multiple comparisons was applied.

In participants with dementia, typically, high cognitive reserve (in those with high education and/or occupational position) is depleted to a great extent, as often reflected in greater neural loss and accelerated decline compared to patients with dementia with low reserve (Stern, 2002, 2009; Stern et al., 2020). This is described as a compensatory effect, where individuals with high reserve can compensate for neurodegeneration by using cognitive reserve related resources until these are no longer available due to overwhelming neuropathology, when dementia becomes clinically apparent. We found some evidence for this pre-dementia compensatory effect of cognitive reserve, whereby hippocampal volume and total gray matter volume were more beneficial to visuospatial skills among those with less education (Table 4). This may suggest that, after dementia diagnosis, individuals with low (but not high) education can still compensate for advancing neuropathology and perform relatively well when neural resources are available and poorly when they are not. In fact, illustration of the effects estimated separately for low and high education (Figure 2A) suggested that there was a negative association between greater hippocampal volume and poorer visuospatial skills for those with higher education specifically. This effect may be spurious as we would more likely expect a null effect and, in fact, these effects were reduced to null after correction for multiple comparisons. Alternatively, it may be that other biological factors besides hippocampal volume explain this effect. That is, visuospatial performance may be particularly impaired in those with high cognitive reserve (because of more extensive underlying neuropathology at/after dementia onset), but it may not be fully reflected in the measurement of volume alone. Only participants with dementia in the mild to moderate stage of the syndrome were included, where memory impairment dominates. It may be that structures outside of the hippocampus drive differences in visuospatial performance. This explanation is also supported by the fact that the pattern of results seemed to be driven by low education in the total gray matter volume analyses. Age- and disease-related heterogeneity in gray matter atrophy exists (Fjell et al., 2014; Kang et al., 2019), so this global measure of brain integrity may reflect distinct atrophy patterns that could differentially influence visuospatial ability. Previously observed interactions between lifestyle factors, early life exposures, and biological mechanisms (Arenaza-Urquijo and Vemuri, 2018; de Rooij, 2022; Hoenig and Drzezga, 2022) indicate that complex interrelations between cognitive reserve and brain volume may result in different groups of reserve driving patterns found in different brain areas.

According to the cognitive reserve hypothesis (Stern, 2009), the relationship between brain integrity and cognitive performance differs depending on the clinical impairment under investigation. Specifically, individuals without cognitive impairment who have higher reserve can rely on preserved resources. Once clinical impairment sets in, there is a transition from still performing well with extensive neuropathology to the same individuals often exhibiting poorer cognitive performance due to greater underlying neuropathology which progressed to the point where cognitive functions are no longer protected. This relationship maps onto the mechanisms through which cognitive reserve is thought to operate—specifically, neural reserve and neural compensation (Stern, 2009).

Our results indicate that for participants without dementia (i.e., SCD and aMCI), (a) greater brain volume, whether hippocampal or total gray matter volume, was relatively consistently associated with better cognitive scores; (b) in terms of cognitive reserve proxies, both higher educational attainment and higher occupational position were related to better attention/working memory, executive control, language, and memory; and (c) the association between greater neural resources (i.e., hippocampal or total gray matter volume) and better cognitive scores was generally stronger among participants with greater cognitive reserve. Results from supplemental analyses restricted to participants with data available on main lifetime occupation were generally consistent with main analyses.

The significant moderations indicating a stronger relationship between brain volume and cognitive abilities for participants without dementia who had higher cognitive reserve suggest that individuals with higher education or occupational position are better capable of utilizing remaining neural resources to preserve cognitive performance. Particularly the moderation by education of the relationship between hippocampal volume and executive control is intriguing and may have important clinical and practical implications. Measures of executive control are reflective of daily functioning (including instrumental and basic activities of daily living; Vaughan and Giovanello, 2010; Martyr and Clare, 2012) and executive dysfunction has been reported in early AD, where hippocampal volume is particularly relevant (Albert, 1996). Therefore, paying attention to markers of cognitive reserve in determining underlying neurodegeneration in relation to the actual clinical diagnosis of dementia may be particularly important, as those with higher reserve may be more likely to be diagnosed later in their progression through the typical AD-related neuropathological spectrum. However, given the cross-sectional nature of this study, we cannot rule out that the patterns of results could be explained by developmental or other pre-existing differences.

Supplemental analyses separating participants without dementia into SCD and aMCI groups revealed similar results to the analyses in which the two groups were combined, although the moderating effects of cognitive reserve on the brain volume-cognition associations appeared to be more apparent in participants with SCD compared to aMCI. This pattern may suggest that cognitive reserve begins to lose some of the protective effects on cognitive performance as individuals progress through stages of cognitive impairment. Additionally, results for SCD were found for both the hippocampal volume analyses and total gray matter volume analyses whereas for aMCI they were found for total gray matter volume analyses only. Since the hippocampus is affected early along the AD continuum, the lack of effects for aMCI may indicate impairment that has started in the hippocampus but has yet to influence other brain areas.

For participants with dementia, the relationship between greater total gray matter volume and better visuospatial skills was magnified in those with less education, providing evidence for the compensatory aspect of cognitive reserve. There are several plausible explanations for this result. First, individuals with high reserve who may have experienced significant atrophy (leading to less objective brain volume) can compensate for this loss and still perform well cognitively. Second, individuals with high reserve who have objectively greater brain volume, but poorer cognitive performance compared to individuals with low reserve, may have already experienced relatively extensive atrophy compared to their pre-dementia volume. Therefore, even though they appear to have greater absolute volume than the sample average, this presumed volume loss may have contributed to their poor performance on cognitive tests relative to participants with low cognitive reserve. These assumptions should be tested with longitudinal data.

Given that visuospatial skills was the only domain that evidenced a significant moderation, it is possible that the neuropathology experienced in dementia may have been too extensive for participants with high reserve to compensate performance in the other cognitive domains. Still, higher education has also been found to strengthen the effect of total gray matter atrophy on cognitive decline (Mungas et al., 2018), so this pattern may reflect the rapid decline participants with high reserve experience after dementia onset. Sensitivity analyses limited to participants with an AD dementia diagnosis (pure or mixed) did not reveal any significant moderating effects of cognitive reserve on the brain volume-cognition relationship. This may be the result of an underpowered analysis, since the magnitude of effects were similar for the interactions found in main analyses, but these effects did not meet the significance criterion.

The supplemental analyses restricting the sample to participants with data on main lifetime occupation revealed relatively consistent results, except that a significant interaction between hippocampal volume and language appeared. This pattern may suggest that language ability is particularly affected by prolonged exposure to a particular work environment.

We also conducted analyses in participants without and with dementia stratified by sex. Overall, regardless of what domain or proxy was of focus, in men and women without dementia, higher cognitive reserve strengthened the brain volume-cognition relationship. There was one overlapping effect between men and women without dementia (i.e., interaction between total gray matter volume and education on language performance). In men, education moderated the total gray matter volume associations with memory and visuospatial skills, and occupational position moderated the total gray matter volume-attention/working memory relationship. In women, education moderated the association between hippocampal volume and executive control, and occupational position moderated the associations between total gray matter volume and executive control, language, and memory. The magnitude of the interaction effects was slightly stronger in women. In participants with dementia, there were no significant effects found for men, but inverse relationships found between brain volume and cognition for women with high education. Future work should assess sex in relation to cognitive reserve to determine whether these effects replicate.

### Interpretation of findings

Our results contribute to the growing field assessing relationships between cognitive reserve, brain health, and cognitive/clinical outcomes by suggesting that both the protective and compensatory components of cognitive reserve seem to be present at different points in clinical impairment. For individuals with higher reserve, there seems to be an inflection point such that before the onset of clinically ascertained dementia, higher reserve is associated with better brain health; cognitive reserve becomes compensatory once neuropathology begins to accumulate allowing individuals to maintain cognitive performance and avoid clinical impairment; and finally individuals with high reserve exhibit accelerated cognitive decline due to the expansive accumulation of neuropathology over time. Our results provide additional evidence for the notion that the transition from cognitive reserve being protective to compensatory may occur at the clinical onset of dementia (Stern et al., 2020). Although our study does not include measures of neuropathology, neurodegeneration is thought to be a downstream process that results from the accumulation of beta-amyloid and tau in the brain, followed by loss of volume. The role of cognitive reserve as protective vs. compensatory against neuropathology may reflect the same underlying process which should be assessed more thoroughly in future work.

A non-linear relationship between brain health and cognitive outcomes among individuals with high reserve may help explain our results and help disentangle the discrepant findings of past research investigating the moderating effect of cognitive reserve on the brain health-cognition relationship. Staekenborg et al. (2020) recently hypothesized this U-shaped relationship between cognitive reserve and neuropathology which also aligns with the model of compensation Gregory et al. (2017) present to understand relationships between cognitive performance and brain volume in neurodegenerative disease. A U-shaped relationship between cognitive reserve, brain volume, and neuropathology supports both (a) the stronger positive relationship between brain volume and cognition for individuals with high reserve prior to the probable appearance of extensive neuropathology and clinical impairment and (b) the attenuated relationship between brain volume and cognitive outcomes for individuals with high reserve after extensive neuropathology has likely accumulated and the compensatory mechanism of reserve is no longer available. Conversely, regarding (a), given the cross-sectional nature of this study, the positive relationship between brain volume and cognition found in participants without dementia could be due to developmental or other pre-existing differences between groups. Regarding (b), we also found evidence of an inverse relationship between brain volume and cognition for participants with high reserve in the dementia group. This may reflect two complementary phenomena: first, individuals with high reserve who have experienced neurodegeneration may still be able to compensate their performance despite the relatively extensive neurodegeneration. This pattern is reflected in Figure 2 where participants with less brain volume than the sample average, but high cognitive reserve, perform better than participants at the same level of brain volume but who have low cognitive reserve. Second, individuals with greater brain volume relative to the sample average and high cognitive reserve may reflect individuals who have experienced extensive neurodegeneration but still have objectively greater brain volume than the sample, impeding on their ability to perform cognitively at the expected level. Finally, our study includes brain volume rather than neuropathology. Since brain atrophy would be farther down the cascade than neuropathological changes including the spread of beta-amyloid and tau in the brain, it is possible that a different pattern results in this downstream measure compared to more immediate neuropathology effects.

Another explanation of divergent findings could revolve around the inclusion of different types of AD neuropathology or brain integrity in studies. Specifically, what is chosen as the marker of AD neuropathology or brain integrity may impact how cognitive reserve influences the pathology-cognition relationship depending on the level of cognitive impairment of the sample. According to the amyloid-cascade hypothesis (Hardy and Higgins, 1992; Karran et al., 2011; Jack and Holtzman, 2013; Jack et al., 2013), the temporal ordering of AD pathophysiology begins with amyloid-beta, followed by tau, and leading to eventual neurodegeneration. Cognitive reserve’s attenuating effect on neuropathology may be better detected by markers of amyloid-beta accumulation in healthy older adults who exhibit no clinical symptoms of dementia or individuals early in the disease progression before significant cognitive decline occurs (Menardi et al., 2018). Subsequently, later pathophysiological markers of AD (i.e., volumetric measures reflecting neurodegeneration) may have a detectable attenuating effect of cognitive reserve on the brain health-cognition relationship once more advanced clinical impairment has occurred. Prior to the appearance of the compensatory mechanism of reserve, associations between brain health and cognitive outcomes may be stronger for individuals with higher levels of reserve compared to lower levels of reserve, representing the neural reserve component of cognitive reserve (Stern, 2009).

Other reasons for the discrepant findings among studies in this area may reflect differences among samples. Sociocultural differences between samples may reflect inconsistent associations between neuropathology and cognition depending on contextual aspects of cognitive reserve proxies. For example, the qualitative aspects of education present in the United States compared to European nations in the 1900s, differences in mandatory or standard educational requirements, and differences in years of education across samples may all contribute to these mixed findings. Similarly, differences in occupational characteristics across culturally distinct geographic regions or occupational opportunities available in certain historical timepoints may also result in disparate findings. Additionally, differences in risk for cognitive decline and dementia are known to exist, yet little is known about how cognitive reserve may relate to these demographic differences. For example, some research suggests that cognitive reserve may operate differently in men and women due to biological or sociocultural differences between the sexes (Rocca, 2017; Ewers, 2020; Subramaniapillai et al., 2021). Investigating these potentially interacting causes of mixed findings is important for future work to better understand what influences individuals’ risk for future impairment.

### Strengths, limitations, and future directions

Strengths of the current study include (a) examination of the cognitive reserve hypothesis among different diagnostic groups to assess how cognitive reserve may differentially moderate the association between brain volume and cognition, (b) the use of an extensive neuropsychological battery that allowed for investigation of several cognitive domains, and (c) the inclusion of two commonly used proxies of cognitive reserve. One main weakness is the cross-sectional design which prevented (a) investigation of longitudinal change in cognition or brain volume, (b) causal/directional interpretation of the results, and (c) the disentanglement of the role of cognitive reserve as protective and/or compensatory. Other weaknesses include: low reliability in one of the cognitive domains (i.e., executive control) as indicated by Cronbach’s alpha values, absence of the use of biomarkers in diagnosis, and the use of a sample from one memory clinic in the Czech Republic which could limit generalizability of results. Future research should include participants who exhibit transitions to dementia from normal cognition to assess how moderating effects of cognitive reserve proxies on brain integrity-cognition relationships may weaken as clinical progression, and the associated neurodegeneration, occurs. For example, testing moderating effects of cognitive reserve at different clinical thresholds (i.e., assessing these relationships in the same participants who transition from SCD to aMCI to dementia) would more clearly reveal the extent to which cognitive reserve operates as protective and/or compensatory and how effects differ in each diagnosis. Additionally, further examination of how development versus brain atrophy may influence associations is warranted. Future work should also consider how inclusion of different AD biomarkers and markers of neuropathology may affect how cognitive reserve moderates associations between brain structure and cognitive outcomes. Finally, since our study presents exploratory findings regarding the moderating effect of cognitive reserve in participants with dementia of different etiologies, future research should isolate AD dementia more specifically to verify the robustness of findings.

### Implications

By recognizing that cognitive reserve moderates the brain volume-cognition relationship, clinical decisions should incorporate markers of cognitive reserve, which in turn may help clinicians identify the link between the level of brain health and actual cognitive performance. If individuals are known to have low markers for reserve (e.g., low education or occupational position), other proxies of reserve could be offered to these individuals (e.g., increased cognitive or physical leisure activity) to promote brain health. At a population-level, more support could be provided for educational attainment and occupational advancement to promote healthy cognitive aging throughout society.

## Conclusion

We provided evidence for cognitive reserve as a moderator of the relationship between brain volume and cognition. This study contributes to the growing evidence that life-course cognitive reserve proxies such as educational attainment and occupational position may play an important role in understanding the association between neural resources, represented by brain volume, and cognition in older adults. The findings also provide unique and new information about the distinct influences of cognitive reserve proxies on the specific associations between brain volume and cognition in participants without and with dementia syndrome.

## Data availability statement

The data analyzed in this study is subject to the following licenses/restrictions: Data available to researchers upon request after completing data use agreement. Requests to access these datasets should be directed to JH, jakub.hort@fnmotol.cz.

## Ethics statement

The studies involving human participants were reviewed and approved by the Institutional Review Board of Motol University Hospital. The patients/participants provided their written informed consent to participate in this study.

## Author contributions

MN: conceptualization, formal analysis, visualization, writing (original draft), and writing (reviewing and editing). BV: conceptualization, formal analysis, and writing (reviewing and editing). RA: conceptualization, formal analysis, supervision, and writing (reviewing and editing). JM and KV: conceptualization, data curation, methodology, and writing (reviewing and editing). HH, ZN, JL, and MV: conceptualization, data curation, funding acquisition, methodology, and writing (reviewing and editing). JH: conceptualization, data curation, funding acquisition, methodology, project administration, and writing (reviewing and editing). All authors contributed to the article and approved the submitted version.

## Footnotes

1
http://surfer.nmr.mgh.harvard.edu

## References

[B1] Albert M. S. (1996). Cognitive and neurobiologic markers of early Alzheimer disease. *Proc. Natl. Acad. Sci. U.S.A.* 93 13547–13551. 10.1073/pnas.93.24.13547 8942970 PMC33644

[B2] Alzheimer’s Association (2022). *What is Alzheimer’s Disease [Online].* Available online at: https://www.alz.org/alzheimers-dementia/what-is-alzheimers (accessed August 1, 2022).

[B3] American Psychiatric Association (2000). *Diagnostic and statistical manual of mental disorders: DSM-IV-TR.* Washington, DC: American Psychiatric Association.

[B4] Arenaza-Urquijo E. M. Vemuri P. (2018). Resistance vs resilience to Alzheimer disease: Clarifying terminology for preclinical studies. *Neurology* 90 695–703. 10.1212/wnl.0000000000005303 29592885 PMC5894932

[B5] Benedict R. H. B. Schretlen D. Groninger L. Dobraski M. Shpritz B. (1996). Revision of the brief visuospatial memory test: Studies of normal performance, reliability, and validity. *Psychol. Assess.* 8 145–153. 10.1037/1040-3590.8.2.145

[B6] Bezdicek O. Motak L. Axelrod B. N. Preiss M. Nikolai T. Vyhnalek M. (2012). Czech version of the trail making test: Normative data and clinical utility. *Arch. Clin. Neuropsychol.* 27 906–914. 10.1093/arclin/acs084 23027441

[B7] Bezdicek O. Stepankova H. Motak L. Axelrod B. N. Woodard J. L. Preiss M. (2014). Czech version of rey auditory verbal learning test: Normative data. *Aging Neuropsychol. Cogn.* 21 693–721. 10.1080/13825585.2013.865699 24344673

[B8] Butters M. A. Young J. B. Lopez O. Aizenstein H. J. Mulsant B. H. Reynolds C. F. III (2008). Pathways linking late-life depression to persistent cognitive impairment and dementia. *Dialogues Clin. Neurosci.* 10 345–357. 10.31887/DCNS.2008.10.3/mabutters 18979948 PMC2872078

[B9] de Rooij S. R. (2022). Are brain and cognitive reserve shaped by early life circumstances? *Front. Neurosci.* 16:825811. 10.3389/fnins.2022.825811 35784851 PMC9243389

[B10] Ewers M. (2020). Reserve in Alzheimer’s disease: Update on the concept, functional mechanisms and sex differences. *Curr. Opin. Psychiatry* 33 178–184. 10.1097/yco.0000000000000574 31789678

[B11] Fischl B. Salat D. H. Busa E. Albert M. Dieterich M. Haselgrove C. (2002). Whole brain segmentation: Automated labeling of neuroanatomical structures in the human brain. *Neuron* 33 341–355. 10.1016/s0896-6273(02)00569-x 11832223

[B12] Fjell A. M. McEvoy L. Holland D. Dale A. M. Walhovd K. B. (2014). What is normal in normal aging? Effects of aging, amyloid and Alzheimer’s disease on the cerebral cortex and the hippocampus. *Prog. Neurobiol.* 117 20–40. 10.1016/j.pneurobio.2014.02.004 24548606 PMC4343307

[B13] Goodglass H. Kaplan E. Weintraub S. (1983). *Boston naming test.* Washington, D.C: Lea & Febiger.

[B14] Gregory S. Long J. D. Klöppel S. Razi A. Scheller E. Minkova L. (2017). Operationalizing compensation over time in neurodegenerative disease. *Brain* 140 1158–1165. 10.1093/brain/awx022 28334888 PMC5382953

[B15] Hardy J. A. Higgins G. A. (1992). Alzheimer’s disease: The amyloid cascade hypothesis. *Science* 256 184–185. 10.1126/science.1566067 1566067

[B16] Hayes A. F. Little T. D. (2018). *Introduction to mediation, moderation, and conditional process analysis: A regression-based approach.* New York, NY: Guilford Publications.

[B17] Hoenig M. C. Drzezga A. (2022). Clear-headed into old age: Resilience and resistance against brain aging-A PET imaging perspective. *J. Neurochem.* 1–21. 10.1111/jnc.15598 35226362

[B18] Holm S. (1979). A simple sequentially rejective multiple test procedure. *Scand. J. Stat.* 6 65–70.

[B19] International Labour Office (2012). *International standard classification of occupations 2008 (ISCO-08): Structure, group definitions and correspondence tables.* Geneva: International Labour Office.

[B20] Jack C. R. Jr. Holtzman D. M. (2013). Biomarker modeling of Alzheimer’s disease. *Neuron* 80 1347–1358. 10.1016/j.neuron.2013.12.003 24360540 PMC3928967

[B21] Jack C. R. Jr. Knopman D. S. Jagust W. J. Petersen R. C. Weiner M. W. Aisen P. S. (2013). Tracking pathophysiological processes in Alzheimer’s disease: An updated hypothetical model of dynamic biomarkers. *Lancet Neurol.* 12 207–216. 10.1016/s1474-4422(12)70291-0 23332364 PMC3622225

[B22] Jessen F. Amariglio R. E. van Boxtel M. Breteler M. Ceccaldi M. Chetelat G. (2014). A conceptual framework for research on subjective cognitive decline in preclinical Alzheimer’s disease. *Alzheimers Dement* 10 844–852. 10.1016/j.jalz.2014.01.001 24798886 PMC4317324

[B23] Joannette M. Bocti C. Dupont P. S. Lavallée M. M. Nikelski J. Vallet G. T. (2020). Education as a moderator of the relationship between episodic memory and amyloid load in normal aging. *J Gerontol. A Biol. Sci. Med. Sci.* 75 1820–1826. 10.1093/gerona/glz235 31639181 PMC7518567

[B24] Johnson P. O. Fay L. C. (1950). The Johnson-Neyman technique, its theory and application. *Psychometrika* 15 349–367. 10.1007/bf02288864 14797902

[B25] Kang D. W. Lim H. K. Joo S. H. Lee N. R. Lee C. U. (2019). Differential associations between volumes of atrophic cortical brain regions and memory performances in early and late mild cognitive impairment. *Front. Aging Neurosci.* 11:245. 10.3389/fnagi.2019.00245 31551759 PMC6738351

[B26] Karran E. Mercken M. De Strooper B. (2011). The amyloid cascade hypothesis for Alzheimer’s disease: An appraisal for the development of therapeutics. *Nat .Rev. Drug. Discov.* 10 698–712. 10.1038/nrd3505 21852788

[B27] Kerbler G. M. Nedelska Z. Fripp J. Laczó J. Vyhnalek M. Lisý J. (2015). Basal forebrain atrophy contributes to allocentric navigation impairment in Alzheimer’s Disease patients. *Front. Aging Neurosci.* 7:185. 10.3389/fnagi.2015.00185 26441643 PMC4585346

[B28] Lee D. H. Lee P. Seo S. W. Roh J. H. Oh M. Oh J. S. (2019). Neural substrates of cognitive reserve in Alzheimer’s disease spectrum and normal aging. *Neuroimage* 186 690–702. 10.1016/j.neuroimage.2018.11.053 30503934

[B29] Martyr A. Clare L. (2012). Executive function and activities of daily living in Alzheimer’s disease: A correlational meta-analysis. *Dement Geriatr. Cogn. Disord.* 33 189–203. 10.1159/000338233 22572810

[B30] Mazancova A. F. Nikolai T. Stepankova H. Kopecek M. Bezdicek O. (2017). The reliability of clock drawing test scoring systems modeled on the normative data in healthy aging and nonamnestic mild cognitive impairment. *Assessment* 24 945–957. 10.1177/1073191116632586 26933141

[B31] McKeith I. G. Boeve B. F. Dickson D. W. Halliday G. Taylor J. P. Weintraub D. (2017). Diagnosis and management of dementia with Lewy bodies: Fourth consensus report of the DLB Consortium. *Neurology* 89 88–100. 10.1212/wnl.0000000000004058 28592453 PMC5496518

[B32] McKhann G. M. Knopman D. S. Chertkow H. Hyman B. T. Jack C. R. Jr. Kawas C. H. (2011). The diagnosis of dementia due to Alzheimer’s disease: Recommendations from the National Institute on Aging-Alzheimer’s Association workgroups on diagnostic guidelines for Alzheimer’s disease. *Alzheimers Dement* 7 263–269. 10.1016/j.jalz.2011.03.005 21514250 PMC3312024

[B33] Menardi A. Pascual-Leone A. Fried P. J. Santarnecchi E. (2018). The role of cognitive reserve in alzheimer’s disease and aging: A Multi-modal imaging review. *J. Alzheimers Dis.* 66 1341–1362. 10.3233/jad-180549 30507572 PMC8972845

[B34] Meyers J. E. Meyers K. R. (1995). *Rey complex figure test and recognition trial (RCFT).* Odessa, FL: Psychological Assessment Resources.

[B35] Michaud T. L. Su D. Siahpush M. Murman D. L. (2017). The risk of incident mild cognitive impairment and progression to dementia considering mild cognitive impairment subtypes. *Dement Geriatr. Cogn. Dis. Extra.* 7 15–29. 10.1159/000452486 28413413 PMC5346939

[B36] Mungas D. Gavett B. Fletcher E. Farias S. T. DeCarli C. Reed B. (2018). Education amplifies brain atrophy effect on cognitive decline: Implications for cognitive reserve. *Neurobiol. Aging* 68 142–150. 10.1016/j.neurobiolaging.2018.04.002 29798764 PMC5993638

[B37] Nedelska Z. Andel R. Laczo J. Vlcek K. Horinek D. Lisy J. (2012). Spatial navigation impairment is proportional to right hippocampal volume. *Proc. Natl. Acad. Sci. U.S.A.* 109 2590–2594. 10.1073/pnas.1121588109 22308496 PMC3289383

[B38] Nikolai T. Stepankova H. Kopecek M. Sulc Z. Vyhnalek M. Bezdicek O. (2018). The uniform data set, czech version: Normative data in older adults from an international perspective. *J. Alzheimers Dis.* 61 1233–1240. 10.3233/JAD-170595 29332045 PMC6939612

[B39] Nikolai T. Štěpánková H. Michalec J. Bezdíček O. Horáková K. Marková H. (2015). Testy verbální fluence, èeská normativní studie pro osoby vyššího věku. *Česká a slovenská neurologie a neurochirurgie* 111 292–299.

[B40] O’Shea D. M. Langer K. Woods A. J. Porges E. C. Williamson J. B. O’Shea A. (2018). Educational attainment moderates the association between hippocampal volumes and memory performances in healthy older adults. *Front. Aging Neurosci.* 10:361. 10.3389/fnagi.2018.00361 30467475 PMC6236013

[B41] Osterrieth P. (1944). Le test de copie d’une figure complexe [The test of copying a complex figure]. *Arch. Psychol.* 30 206–356. 10.1016/j.psychres.2005.10.012 17007938

[B42] Pa J. Aslanyan V. Casaletto K. B. Rentería M. A. Harrati A. Tom S. E. (2022). Effects of sex, APOE4, and lifestyle activities on cognitive reserve in older adults. *Neurology* 99:e789. 10.1212/WNL.0000000000200675 35858818 PMC9484731

[B43] Peltz C. B. Corrada M. M. Berlau D. J. Kawas C. H. (2011). Incidence of dementia in oldest-old with amnestic MCI and other cognitive impairments. *Neurology* 77 1906–1912. 10.1212/WNL.0b013e318238ee89 22076544 PMC3233189

[B44] Petersen R. C. (2004). Mild cognitive impairment as a diagnostic entity. *J. Intern. Med.* 256 183–194. 10.1111/j.1365-2796.2004.01388.x 15324362

[B45] Petersen R. C. Roberts R. O. Knopman D. S. Boeve B. F. Geda Y. E. Ivnik R. J. (2009). Mild cognitive impairment: Ten years later. *Arch. Neurol.* 66 1447–1455. 10.1001/archneurol.2009.266 20008648 PMC3081688

[B46] Rascovsky K. Hodges J. R. Knopman D. Mendez M. F. Kramer J. H. Neuhaus J. (2011). Sensitivity of revised diagnostic criteria for the behavioural variant of frontotemporal dementia. *Brain* 134(Pt 9) 2456–2477. 10.1093/brain/awr179 21810890 PMC3170532

[B47] Rocca W. A. (2017). Time, sex, gender, history, and dementia. *Alzheimer Dis. Assoc. Disord.* 31 76–79. 10.1097/wad.0000000000000187 28169841 PMC5321864

[B48] Román G. C. Tatemichi T. K. Erkinjuntti T. Cummings J. L. Masdeu J. C. Garcia J. H. (1993). Vascular dementia: Diagnostic criteria for research studies. Report of the NINDS-AIREN International Workshop. *Neurology* 43 250–260. 10.1212/wnl.43.2.250 8094895

[B49] Sheardova K. Vyhnalek M. Nedelska Z. Laczo J. Andel R. Marciniak R. (2019). Czech Brain Aging Study (CBAS): Prospective multicentre cohort study on risk and protective factors for dementia in the Czech Republic. *BMJ Open* 9:e030379. 10.1136/bmjopen-2019-030379 31857299 PMC6937049

[B50] Staekenborg S. S. Kelly N. Schuur J. Koster P. Scherder E. Tielkes C. E. M. (2020). Education as proxy for cognitive reserve in a large elderly memory clinic: ‘Window of Benefit’. *J. Alzheimers Dis.* 76 671–679. 10.3233/jad-191332 32538838

[B51] Stern Y. (2002). What is cognitive reserve? Theory and research application of the reserve concept. *J. Intl. Neuropsychol. Soc.* 8 448–460. 11939702

[B52] Stern Y. (2009). Cognitive reserve. *Neuropsychologia* 47 2015–2028. 19467352 10.1016/j.neuropsychologia.2009.03.004 PMC2739591

[B53] Stern Y. Arenaza-Urquijo E. M. Bartrés-Faz D. Belleville S. Cantilon M. Chetelat G. (2020). Whitepaper: Defining and investigating cognitive reserve, brain reserve, and brain maintenance. *Alzheimers Dement* 16 1305–1311. 10.1016/j.jalz.2018.07.219 30222945 PMC6417987

[B54] Subramaniapillai S. Almey A. Natasha Rajah M. Einstein G. (2021). Sex and gender differences in cognitive and brain reserve: Implications for Alzheimer’s disease in women. *Front. Neuroendocrinol.* 60:100879. 10.1016/j.yfrne.2020.100879 33137359

[B55] Sundermann E. E. Maki P. M. Rubin L. H. Lipton R. B. Landau S. Biegon A. (2016). Female advantage in verbal memory: Evidence of sex-specific cognitive reserve. *Neurology* 87 1916–1924. 10.1212/wnl.0000000000003288 27708128 PMC5100712

[B56] Vaughan L. Giovanello K. (2010). Executive function in daily life: Age-related influences of executive processes on instrumental activities of daily living. *Psychol. Aging* 25 343–355. 10.1037/a0017729 20545419

[B57] Voevodskaya O. Simmons A. Nordenskjöld R. Kullberg J. Ahlström H. Lind L. (2014). The effects of intracranial volume adjustment approaches on multiple regional MRI volumes in healthy aging and Alzheimer’s disease. *Front. Aging Neurosci.* 6:264. 10.3389/fnagi.2014.00264 25339897 PMC4188138

[B58] Wechsler D. (1997). *WAiS-iii.* San Antonio, TX: Psychological Corporation.

[B59] Yesavage J. A. Brink T. L. Rose T. L. Lum O. Huang V. Adey M. (1982). Development and validation of a geriatric depression screening scale: A preliminary report. *J. Psychiatr. Res.* 17 37–49. 7183759 10.1016/0022-3956(82)90033-4

